# Supplementary material for: Encapsulated CdSe/CdS nanorods in double-shelled porous nanocomposites for efficient photocatalytic CO2 reduction
Source: Nat Commun. 2022 Oct 29;13:6466. doi: 10.1038/s41467-022-34263-z (PMC9617972; doi:10.1038/s41467-022-34263-z)
Supplement: Supplementary file 1 — Supplementary Information [file 41467_2022_34263_MOESM1_ESM.pdf]

Supplementary Information for

Encapsulated CdSe/CdS nanorods in double-shelled porous nanocomposites for efficient photocatalytic CO_2 reduction

Hui Li[†], Caikun Cheng[†], Zhijie Yang[†], Jingjing Wei[†]*

[†]Key Laboratory of Colloid and Interface Chemistry, Ministry of Education, School of Chemistry and Chemical Engineering, Shandong University, Jinan 250100, P.R. China.

*Corresponds to weijingjing@sdu.edu.cn

Table of contents

1. Supplementary Figures..S3

2. Supplementary Tables...S37

3. Supplementary Notes and Methods S41

1. Supplementary Figures

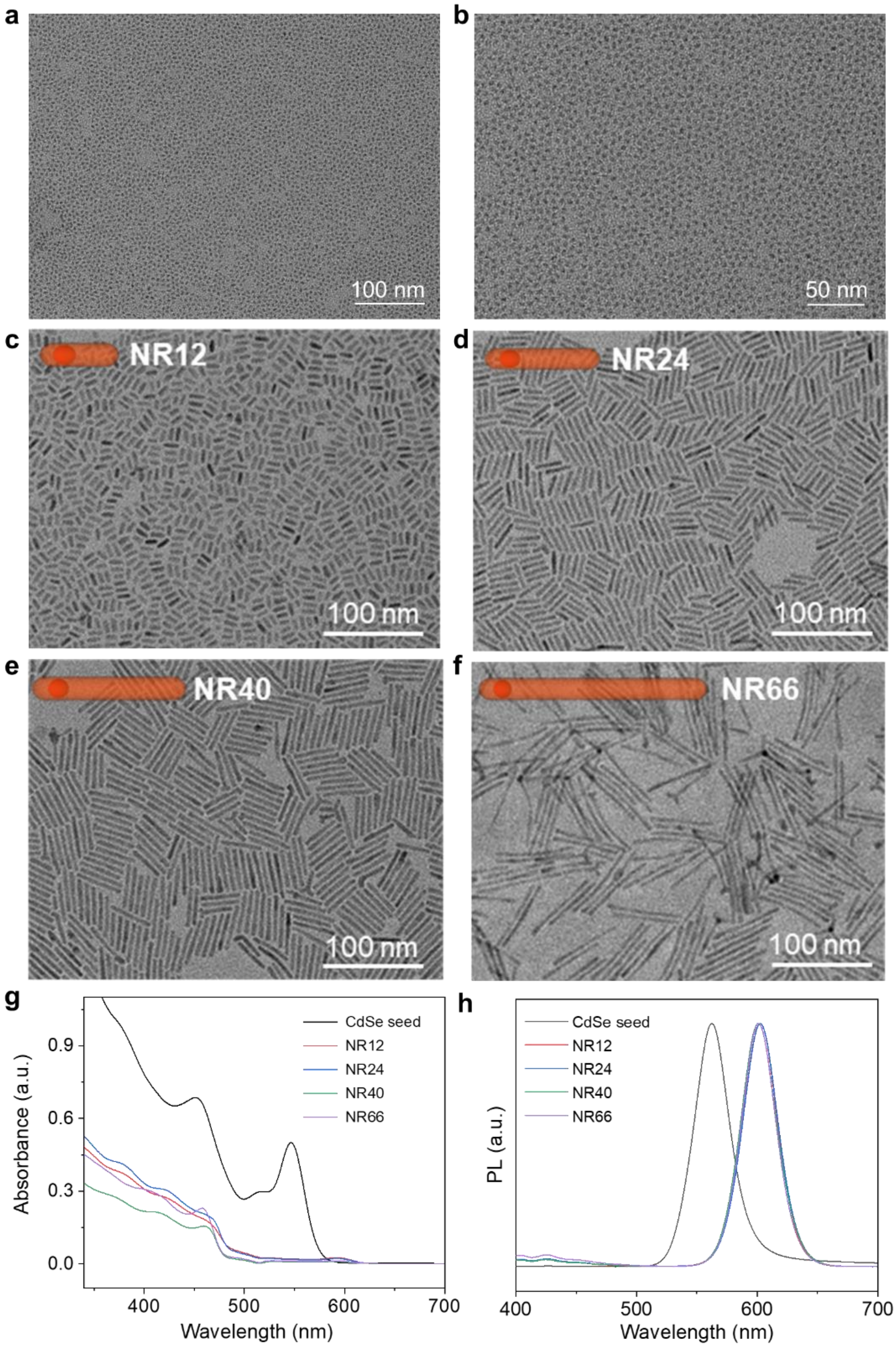

Supplementary Fig. 1. Characterization of NRs. TEM images of CdSe seed (a, b) and CdSe/CdS nanorods with different length (c-f); UV-vis absorption spectra (g) and PL spectra (h) of CdSe seeds and CdSe/CdS nanorods with different lengths.

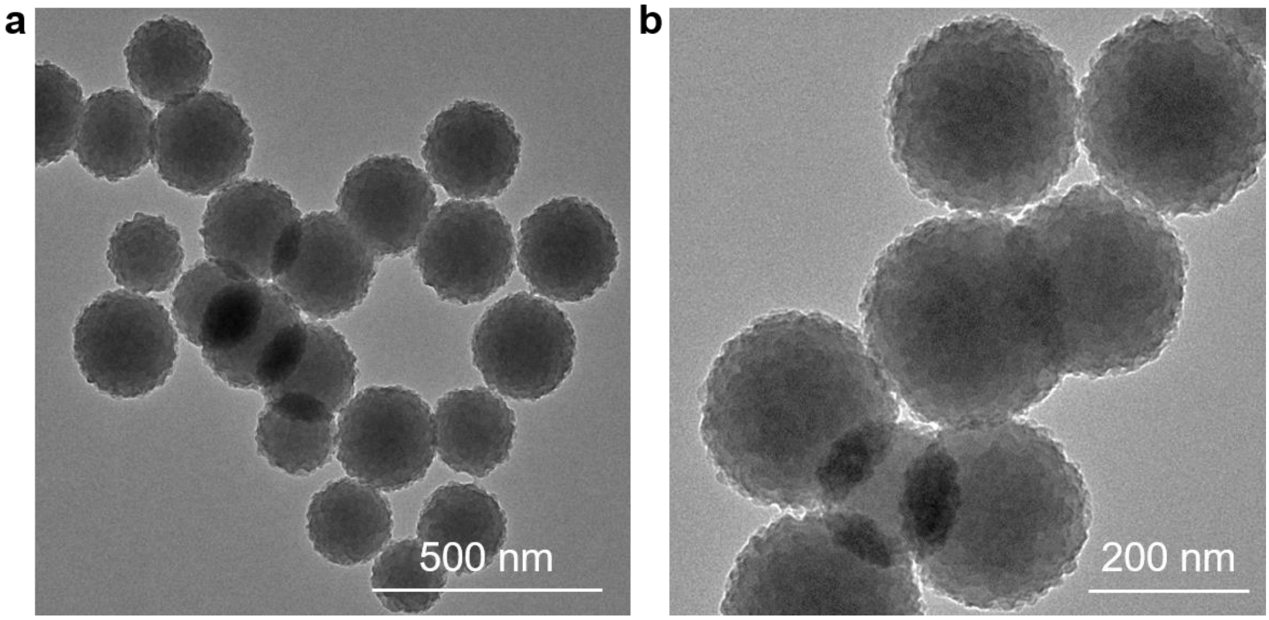

Supplementary Fig. 2. (a,b) TEM images of TAPT-DMTA colloids before crystallization.

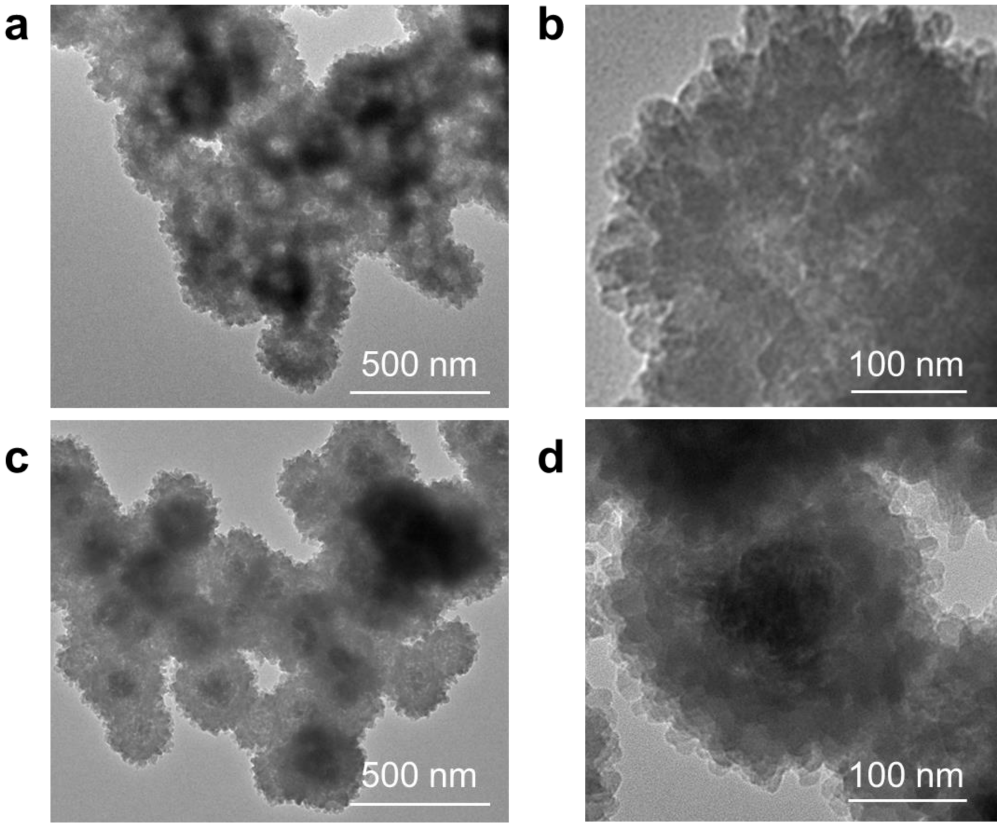

Supplementary Fig. 3. Characterization of the TAPT-DMTA-S and the TAPT-DMTA/NR40-S. TEM images of **TAPT-DMTA-S** (a, b) and **TAPT-DMTA/NR40-S** (c, d).

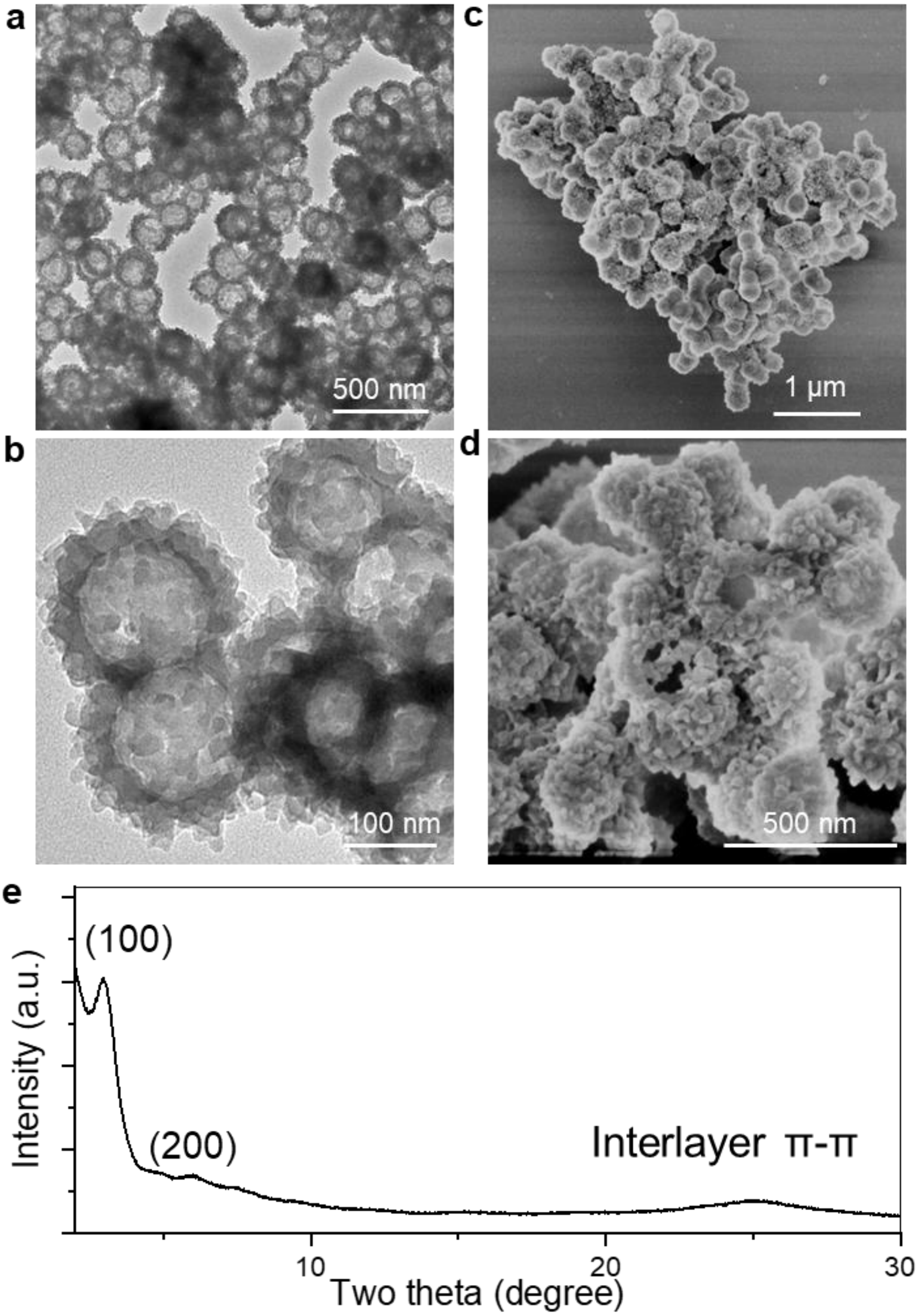

Supplementary Fig. 4. Characterization of the TAPT-DMTA. TEM images (a, b) and SEM images (c, d) of **TAPT-DMTA-H**; The XRD pattern (e) of **TAPT-DMTA-H**.

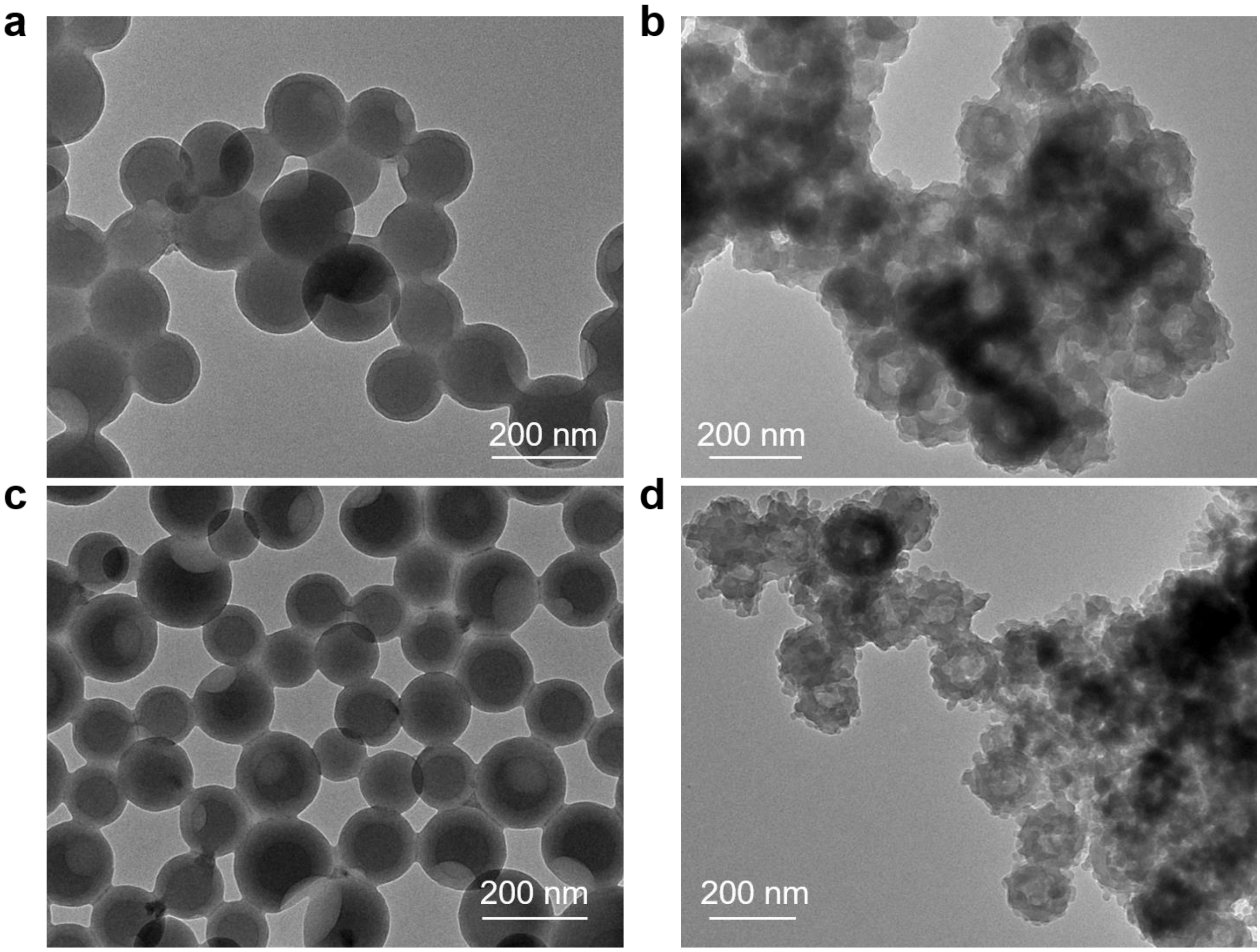

Supplementary Fig. 5. Morphological studies of the TAPA-DMTA and TAPB-DMTA polymers. TEM images of TAPA-DMTA before (a) and after (b) crystallization; TEM images of TAPB-DMTA before (c) and after (d) crystallization.

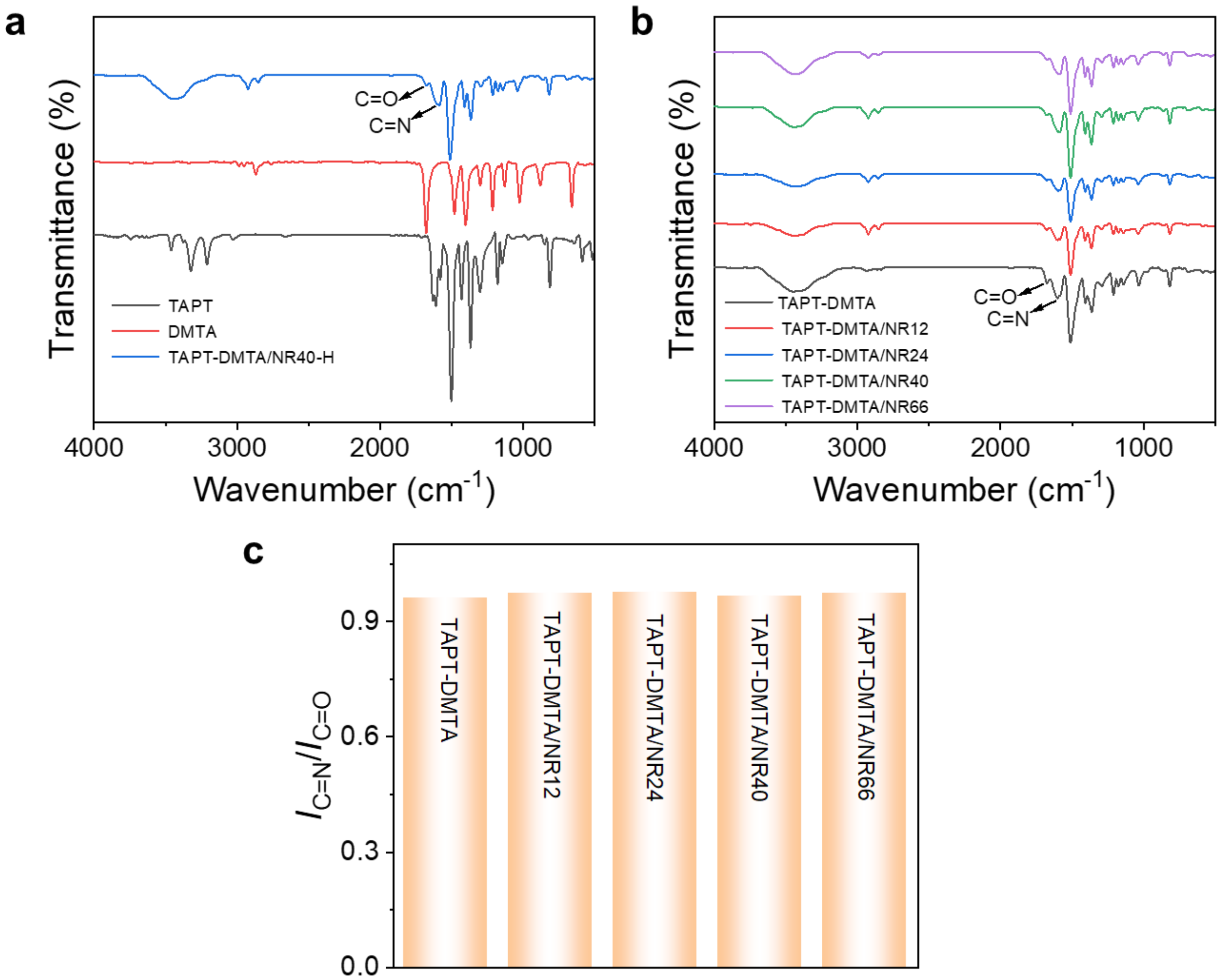

Supplementary Fig. 6. FTIR data of the nanocomposites. (a) FT-IR spectra of TAPT, DMTA and **TAPT-DMTA/NR40-H**; (b) FT-IR spectra of **TAPT-DMTA-H**, **TAPT-DMTA/NR12-H**, **TAPT-DMTA/NR24-H**, **TAPT-DMTA/NR40-H**, **TAPT-DMTA/NR66-H**; (c) $I_{C=N}/I_{C=O}$ ratios of various samples.

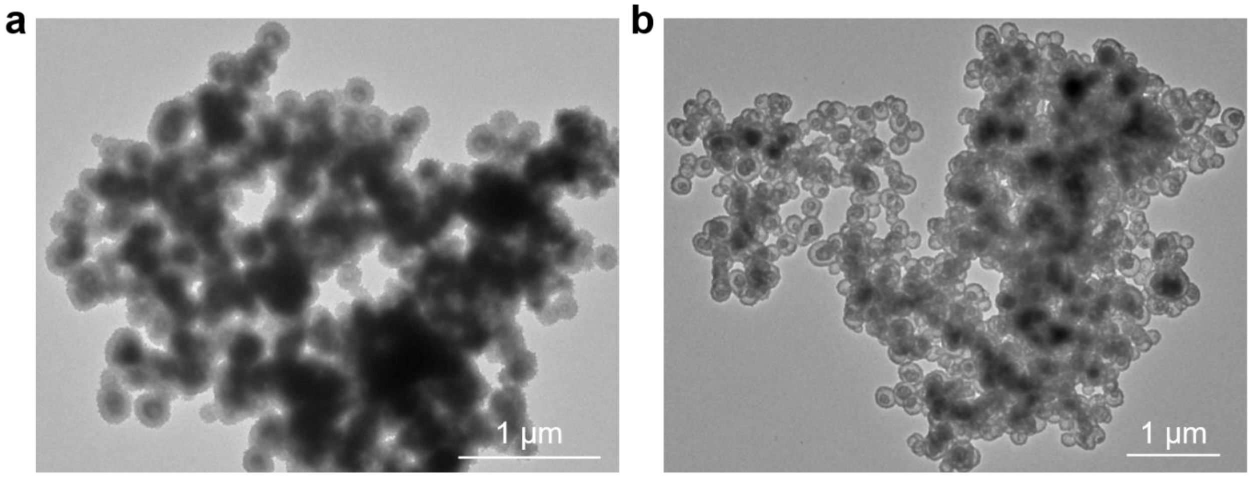

Supplementary Fig. 7. Additional TEM data. (a) TEM image of **TAPT-DMTA/NR40** before crystallization; (b) TEM image of **TAPT-DMTA/NR40** after crystallization.

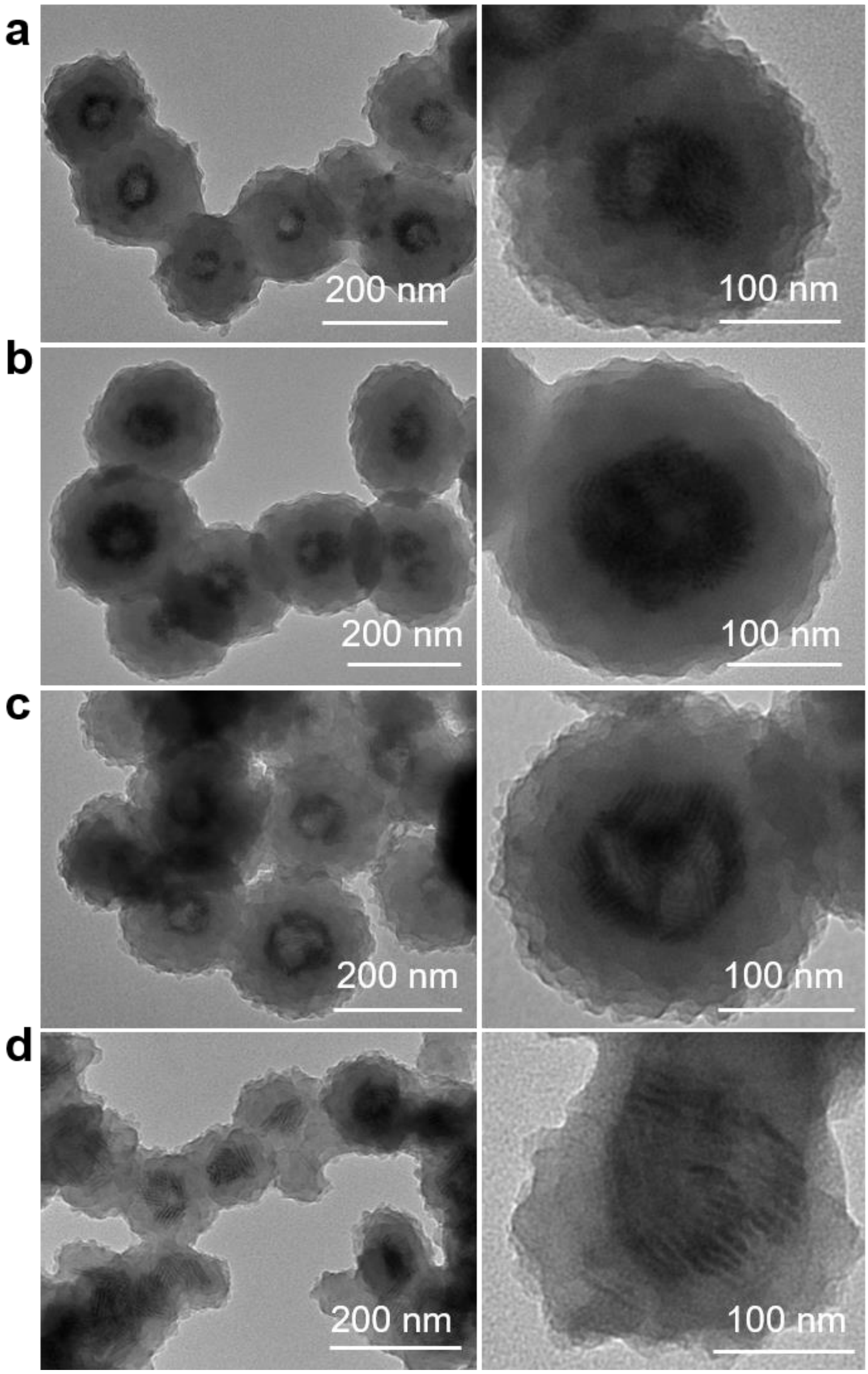

Supplementary Fig. 8. Morphological data of the nanocomposites differing by the length of the NRs before crystallization. (a) TEM images of **TAPT-DMTA/NR12** before crystallization; (b) TEM images of **TAPT-DMTA/NR24** before crystallization; (c) TEM images of **TAPT-DMTA/NR40** before crystallization; (d) TEM images of **TAPT-DMTA/NR66** before crystallization.

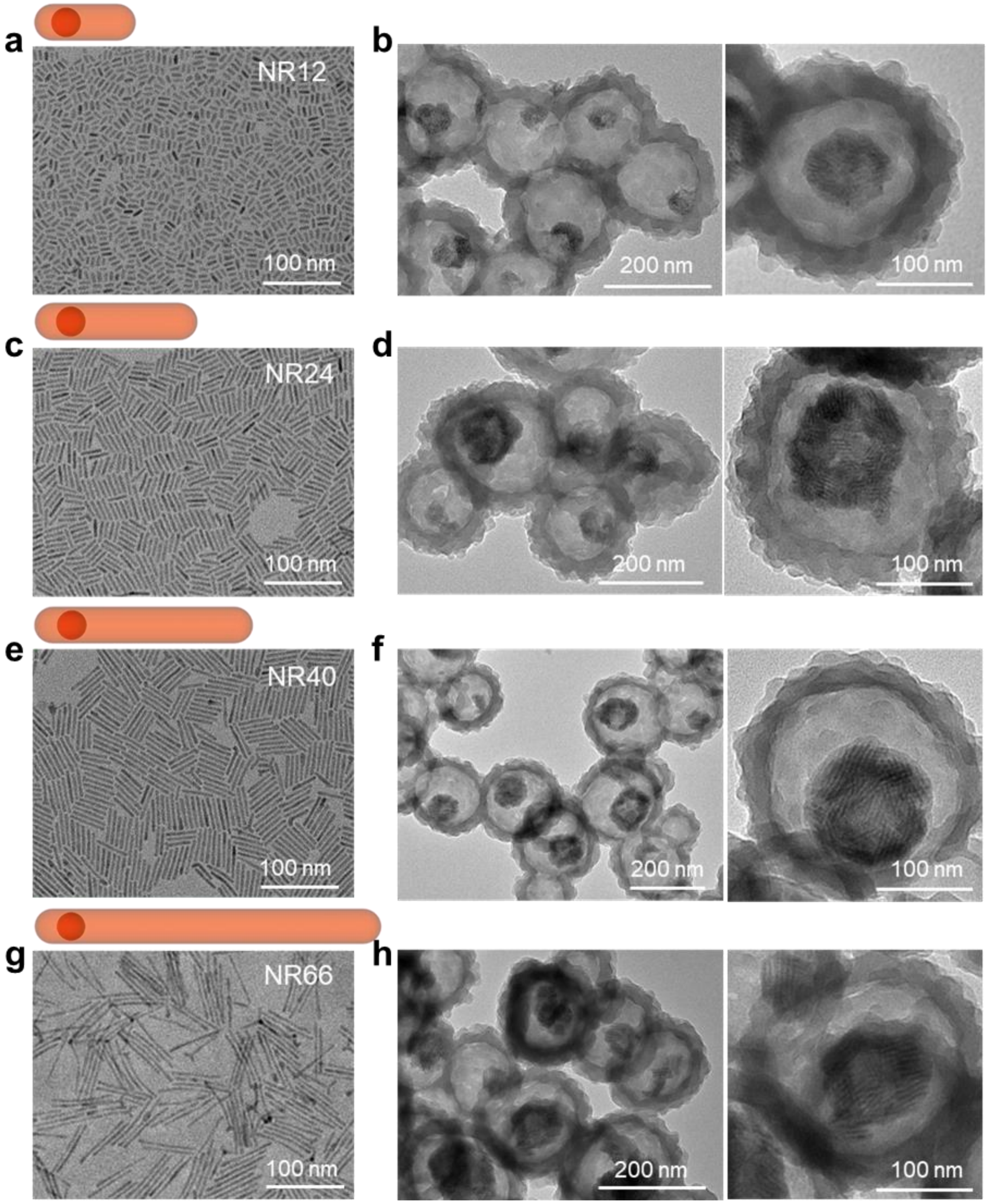

Supplementary Fig. 9. Morphological data of the nanocomposites differing by the length of the NRs. (a) The TEM images of NR12; (b) The TEM images of **TAPT-DMTA/NR12-H**; (c) The TEM images of NR24; (d) The TEM images of **TAPT-DMTA/NR24-H**; (e) The TEM images of NR40; (f) The TEM images of **TAPT-DMTA/NR40-H**; (g) The TEM images of NR66; (h) The TEM images of **TAPT-DMTA/NR66-H**.

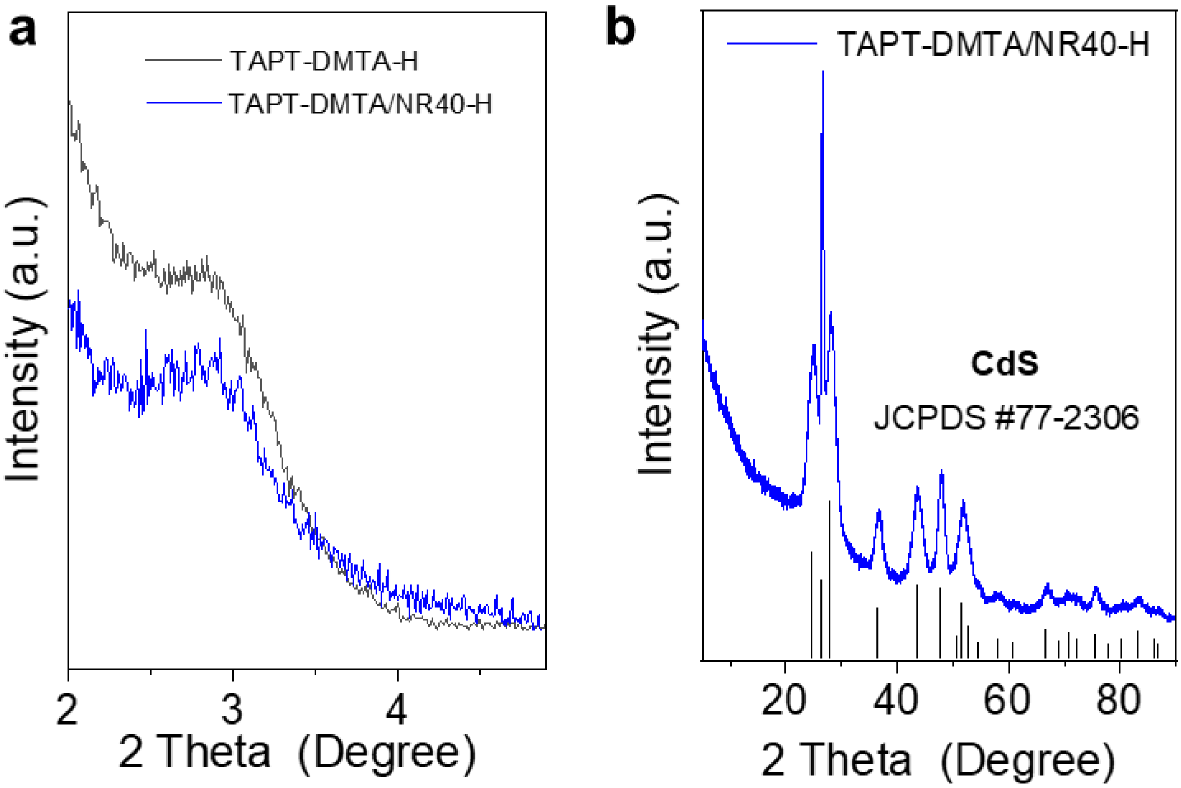

Supplementary Fig. 10. XRD data of the nanocomposites. XRD pattern in small-angle range (a) and wide-angle range (b) of TAPT-DMTA-H and TAPT-DMTA/NR40-H.

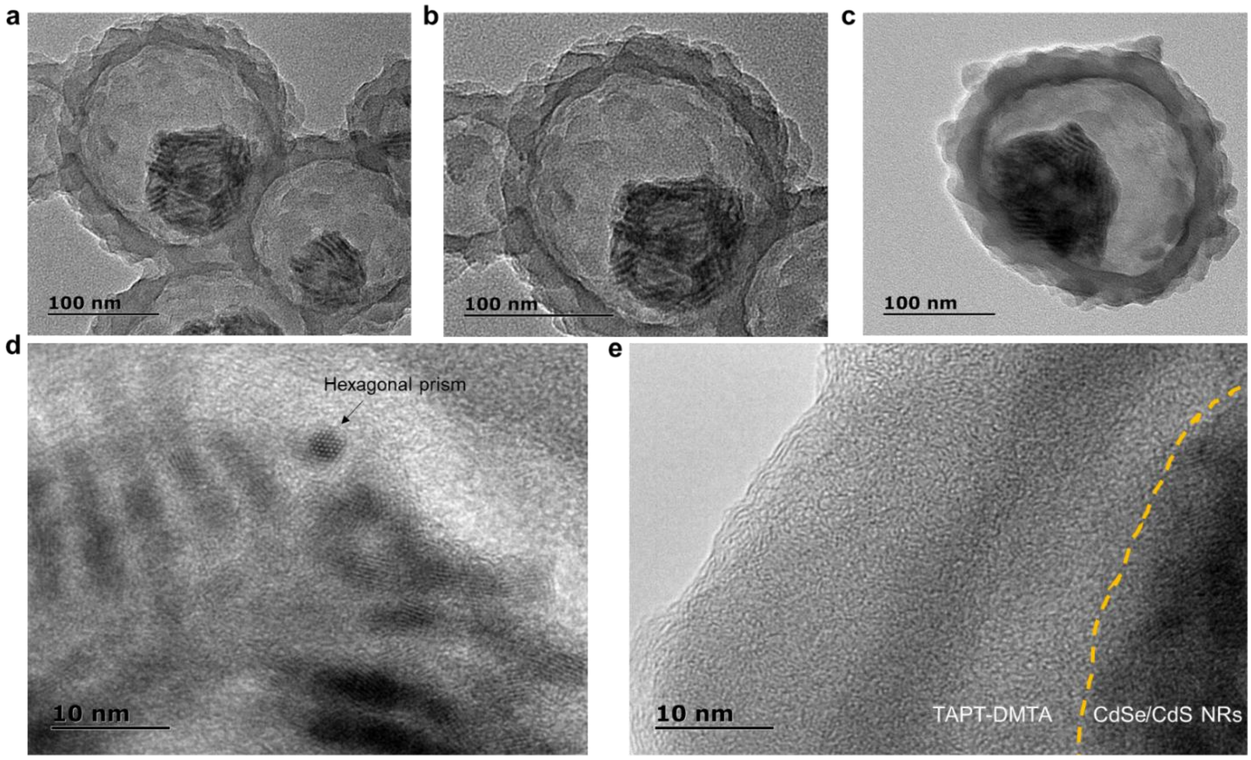

Supplementary Fig. 11. Additional morphological studies on the TAPT-DMTA/NR40-H nanocomposites. HRTEM images of nanocomposites of TAPT-DMTA/NR40-H. (a-c) low magnification TEM images showing the contact between the inner shell of NRs assemblies and the outer shell of TAPT-DMTA polymers; (d-e) high magnification TEM images showing the lattice fringes of the NRs and the boundary between NR assemblies and TAPT-DMTA polymers.

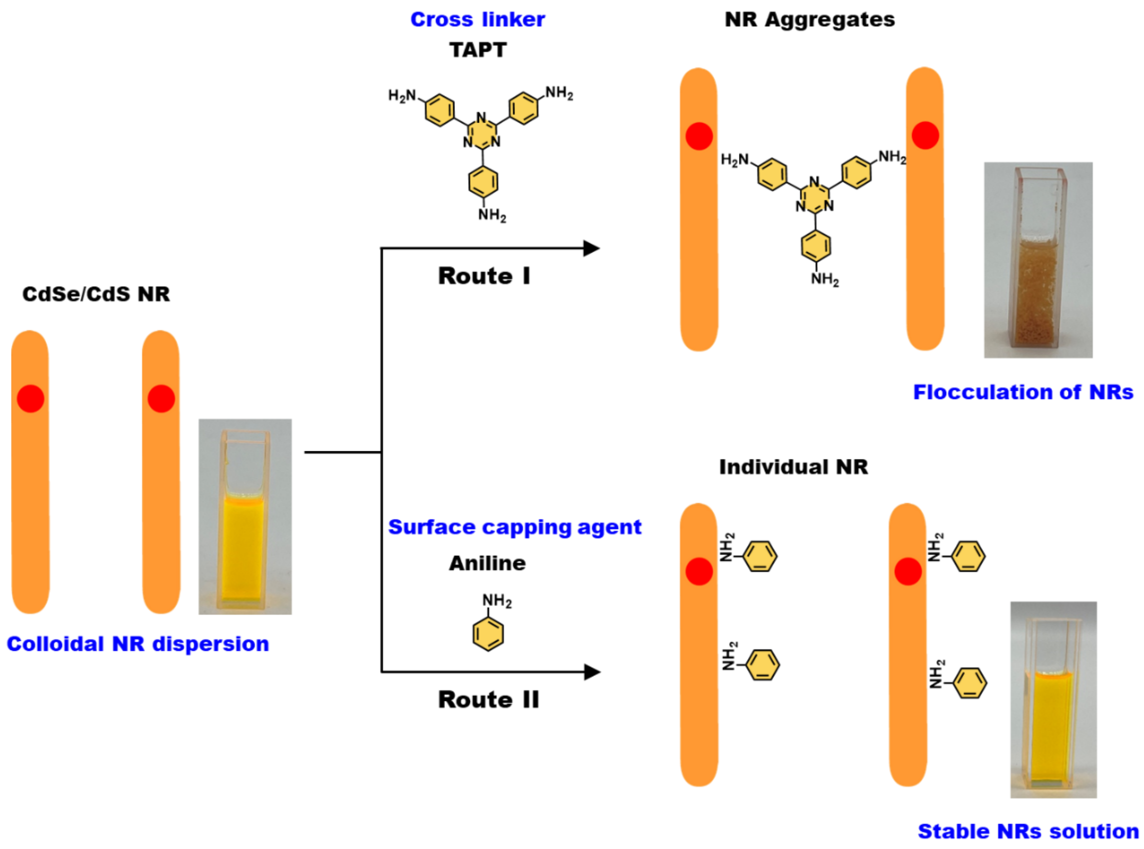

Supplementary Fig. 12. Scheme for additional experiments that confirm the interaction between the amine of COF and the surface of NRs. Two typical routes were applied: Route I and Route II. The native ligands (ODPA/HDA) that capped on the surface of CdSe/CdS NRs were omitted for clarity.

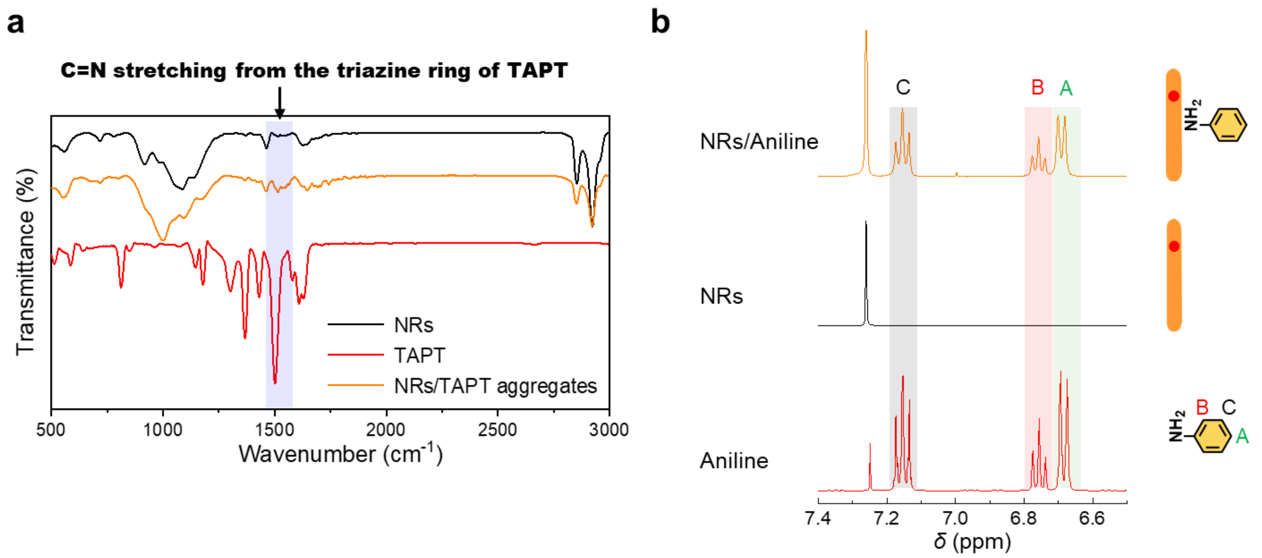

Supplementary Fig. 13. Additional data revealing the interactions between amine and NRs. (a) Infrared spectra of NRs, TAPT, and NRs/TAPT aggregates; (b) ^{1}H NMR spectra of NRs, aniline, and NRs/aniline composites.

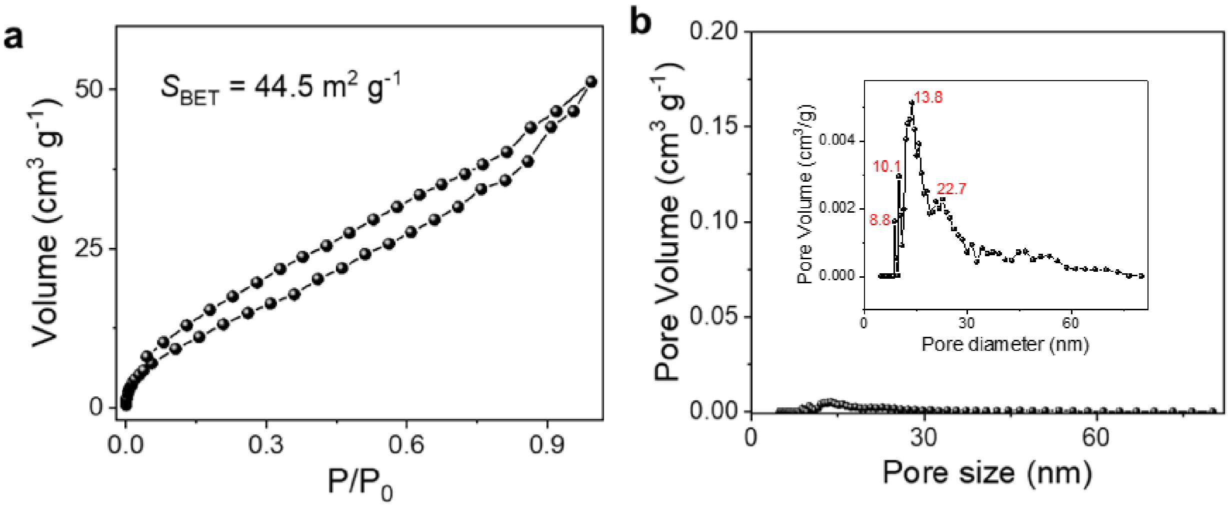

Supplementary Fig. 14. Nitrogen sorption data. The N_2 adsorption-desorption isotherms (a) and pore size distribution (b) of **TAPT-DMTA/NR40** before crystallization.

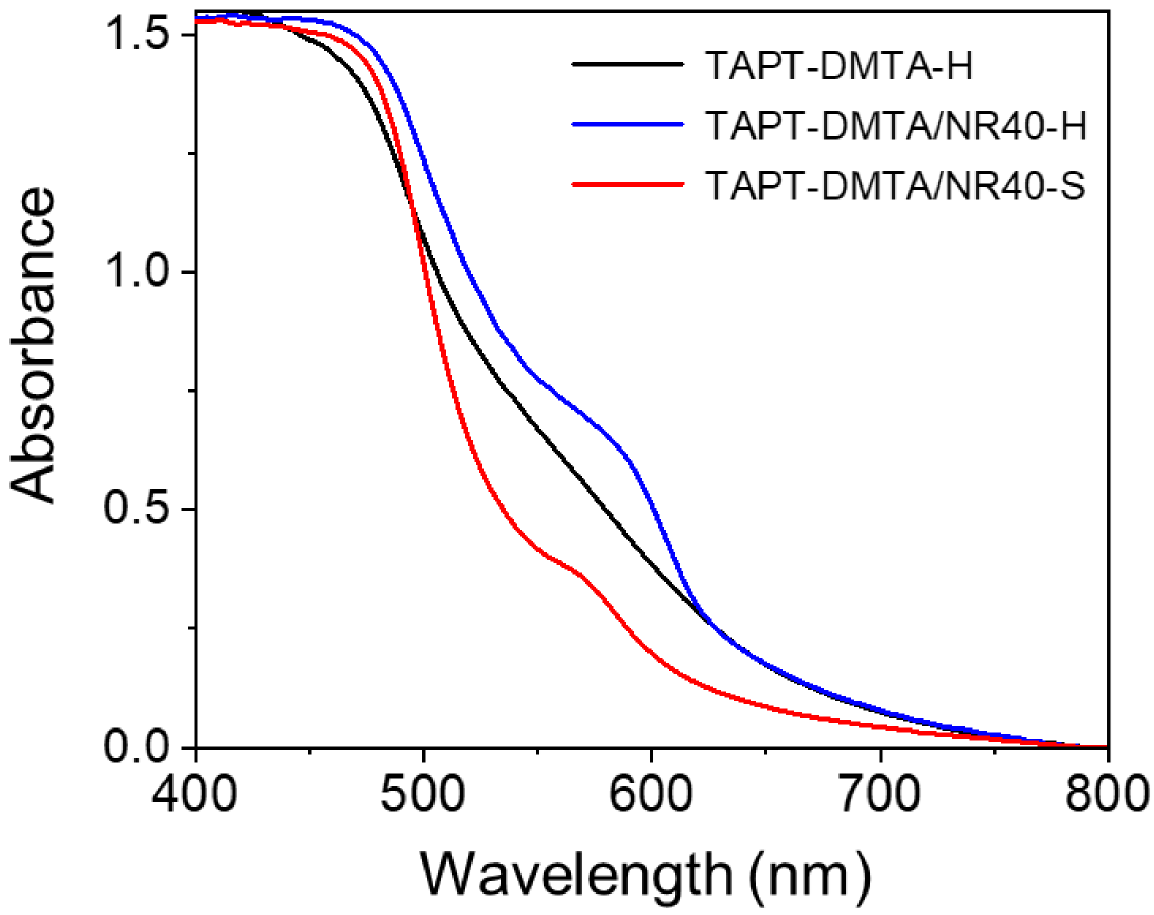

Supplementary Fig. 15. The UV-Vis absorption spectra of **TAPT-DMTA-H**, **TAPT-DMTA/NR40-H** and **TAPT-DMTA/NR40-S** without normalization.

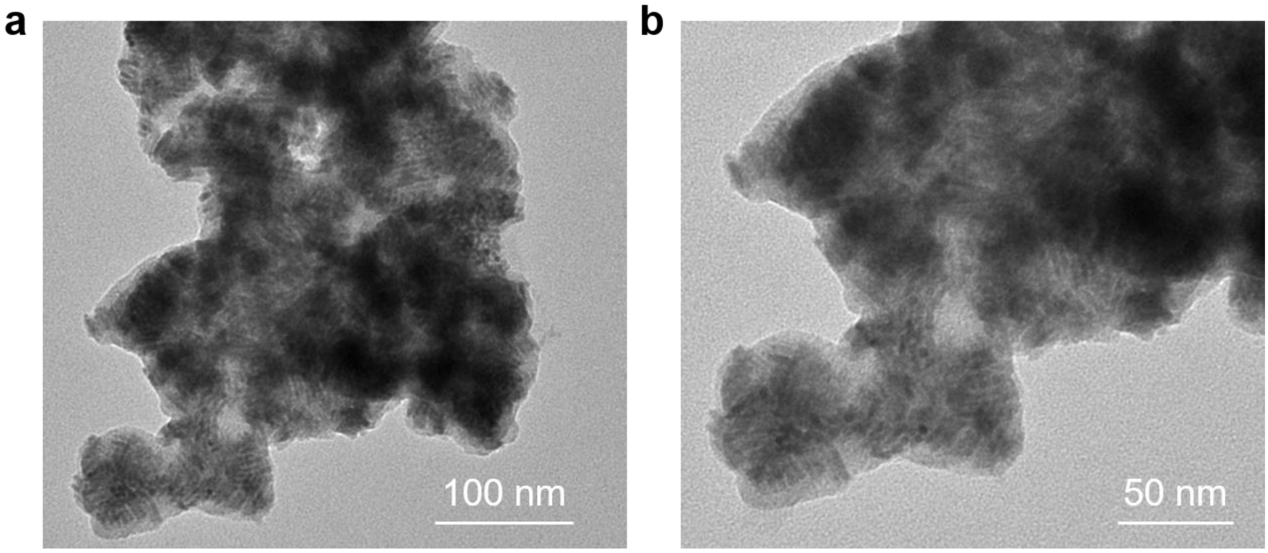

Supplementary Fig. 16. (a, b) TEM images of NR40 assemblies produced without the introduction of organic monomers (TAPT or DMTA).

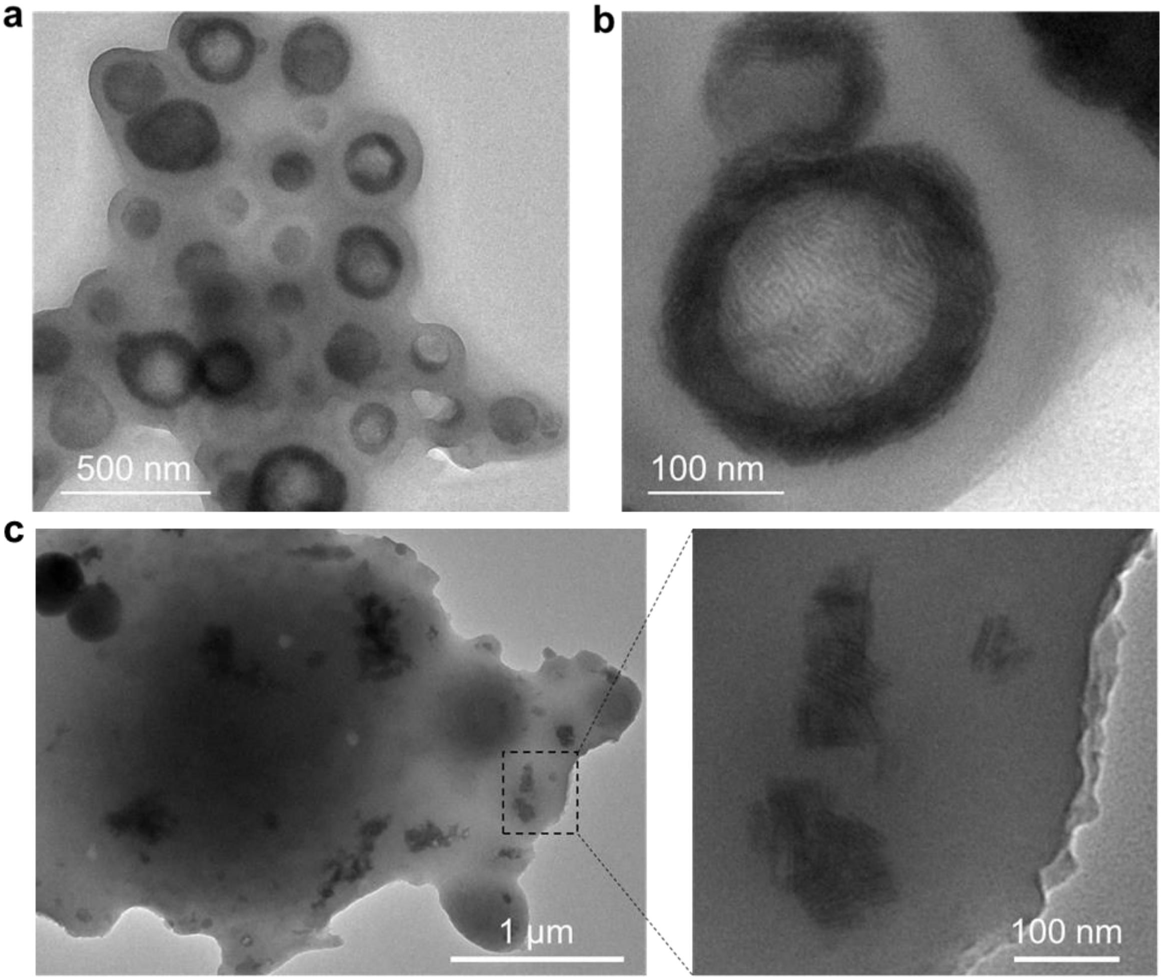

Supplementary Fig. 17. Morphological studies on the nanocomposites from additional control experiments. (a-b) The TEM images of sample in the absence of Lewis acid catalyst; (c) TEM images of nanocomposites of **TAPB-DMTA/NR40** prepared in the absence of DTAB.

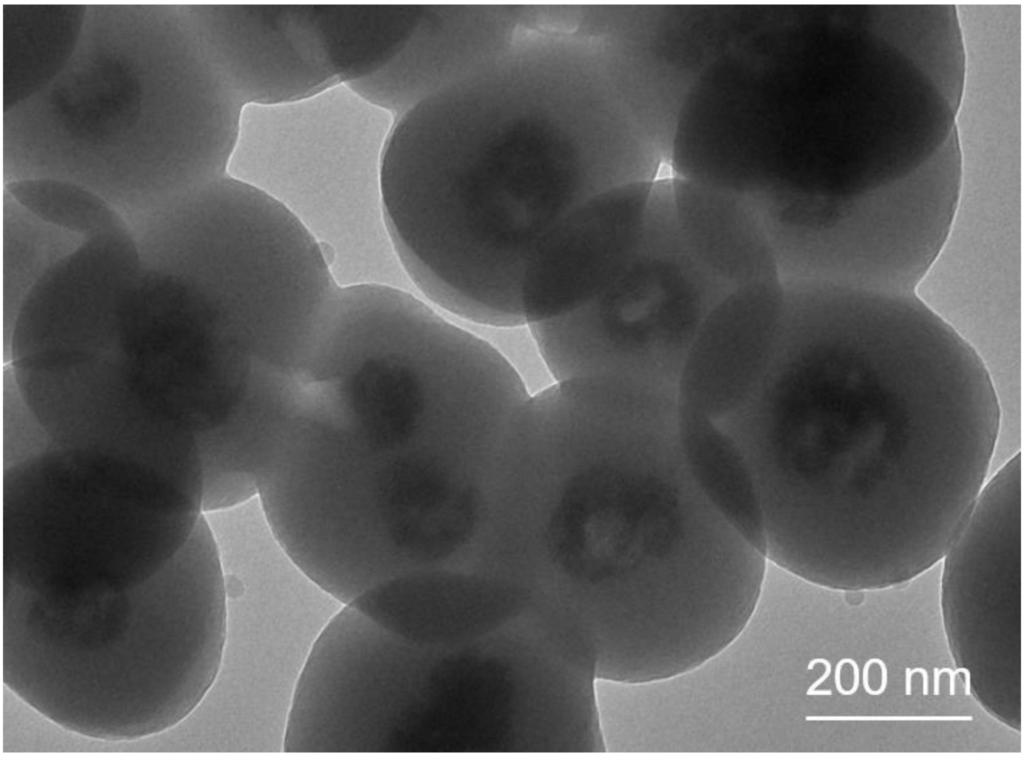

Supplementary Fig. 18. The TEM images of TAPB-DMTA/NR40 emulsified at 50 °C.

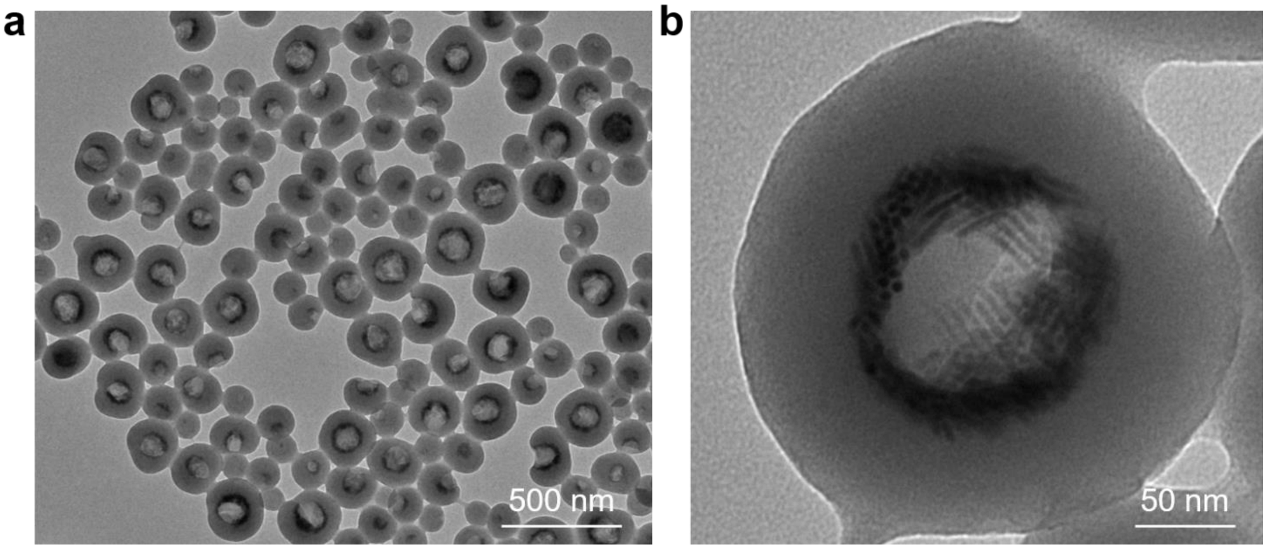

Supplementary Fig. 19. (a, b) TEM images of TAPB-DMTA/NR40 separated immediately after emulsification process.

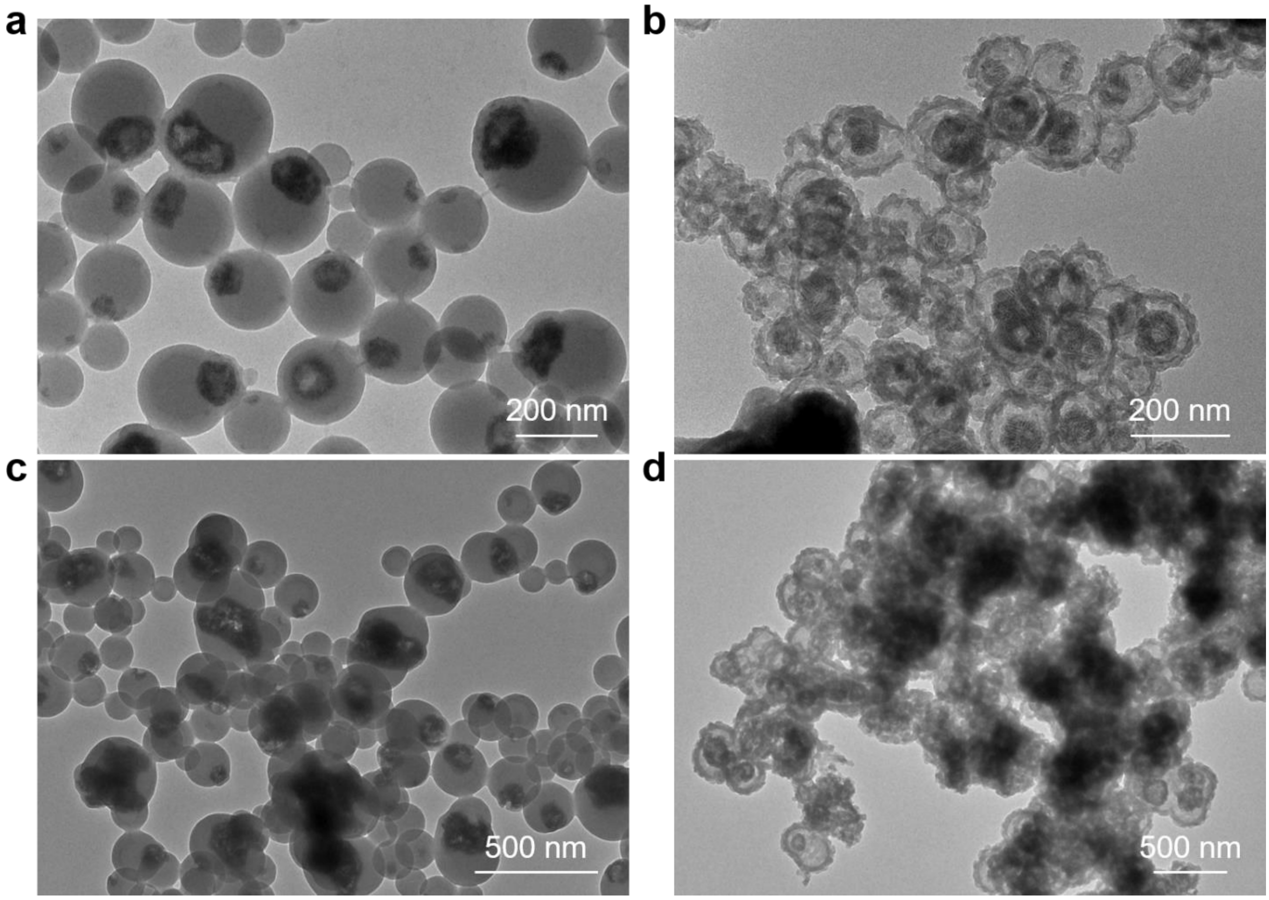

Supplementary Fig. 20. Morphological studies on the nanocomposites. TEM images of TAPA-DMTA/NR40 before and after crystallization (a, b); The TEM images of TAPB-DMTA/NR40 before and after crystallization (c, d).

a

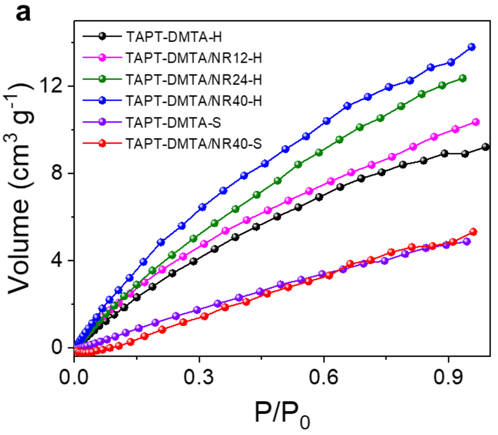

b

CO_2 uptake volume for various samples

Sample	CO_2 uptake volume ($cm^3\ g^{-1}$)
TAPT-DMTA-S	4.9
TAPT-DMTA-H	9.2
TAPT-DMTA/NR40-S	5.3
TAPT-DMTA/NR40-H	13.8
TAPT-DMTA/NR24-H	12.4
TAPT-DMTA/NR12-H	10.4

Supplementary Fig. 21. CO_2 adsorption curves. (a) the CO_2 adsorption curves of various samples at 298 K (b) the summarized CO_2 adsorption values.

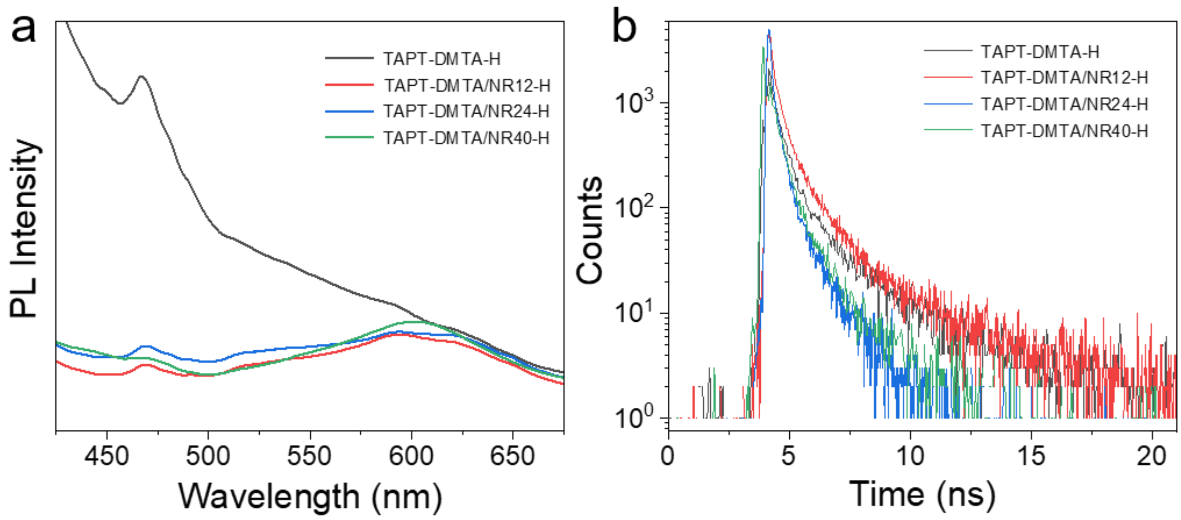

Supplementary Fig. 22. Steady-state and time-resolved PL spectra. Steady-state PL spectra (a) and time resolved PL spectra (b) of TAPT-DMTA-H, TAPT-DMTA/NR12-H, TAPT-DMTA/NR24-H, and TAPT-DMTA/NR40-H.

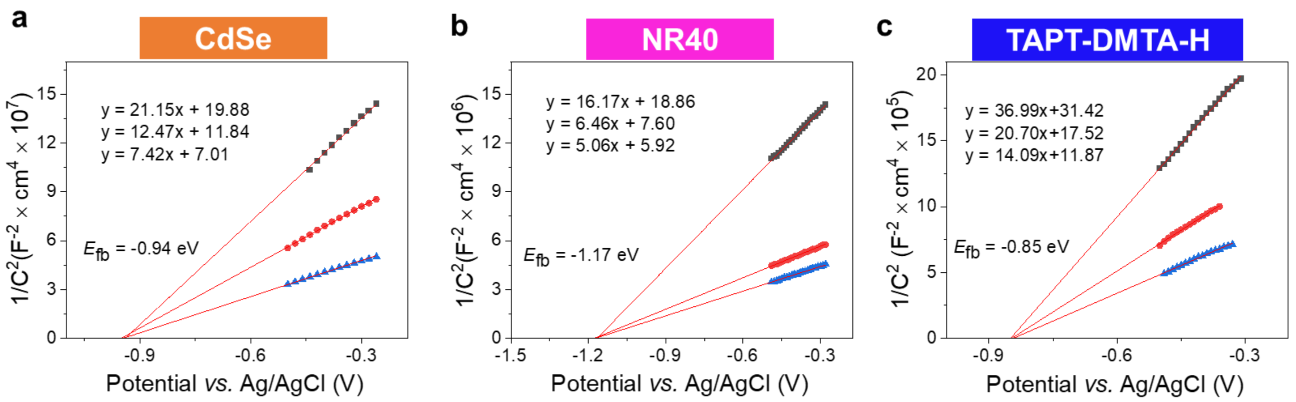

Supplementary Fig. 23. Mott-Schottky plots. Mott-Schottky plots of CdSe seeds (a), CdSe/CdS NR40 (b), and **TAPT-DMTA-H** (c). The blue, red and black curves were measured by applying a frequency of 2000, 1500, and 1000 Hz, respectively. The insets in the plots are the mathematical linear equations.

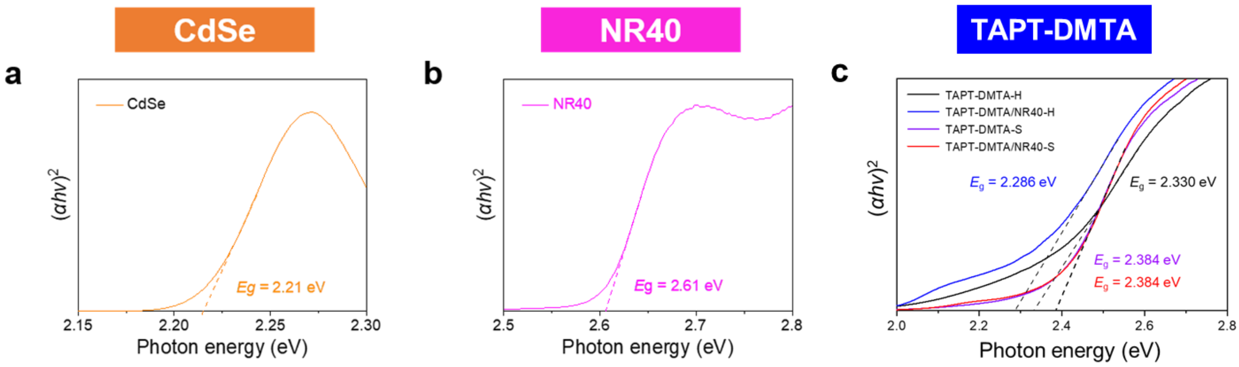

Supplementary Fig. 24. Tauc plots. Tauc plots of CdSe seeds (a), CdSe/CdS NR40 (b) and TAPT-DMTA polymer (c).

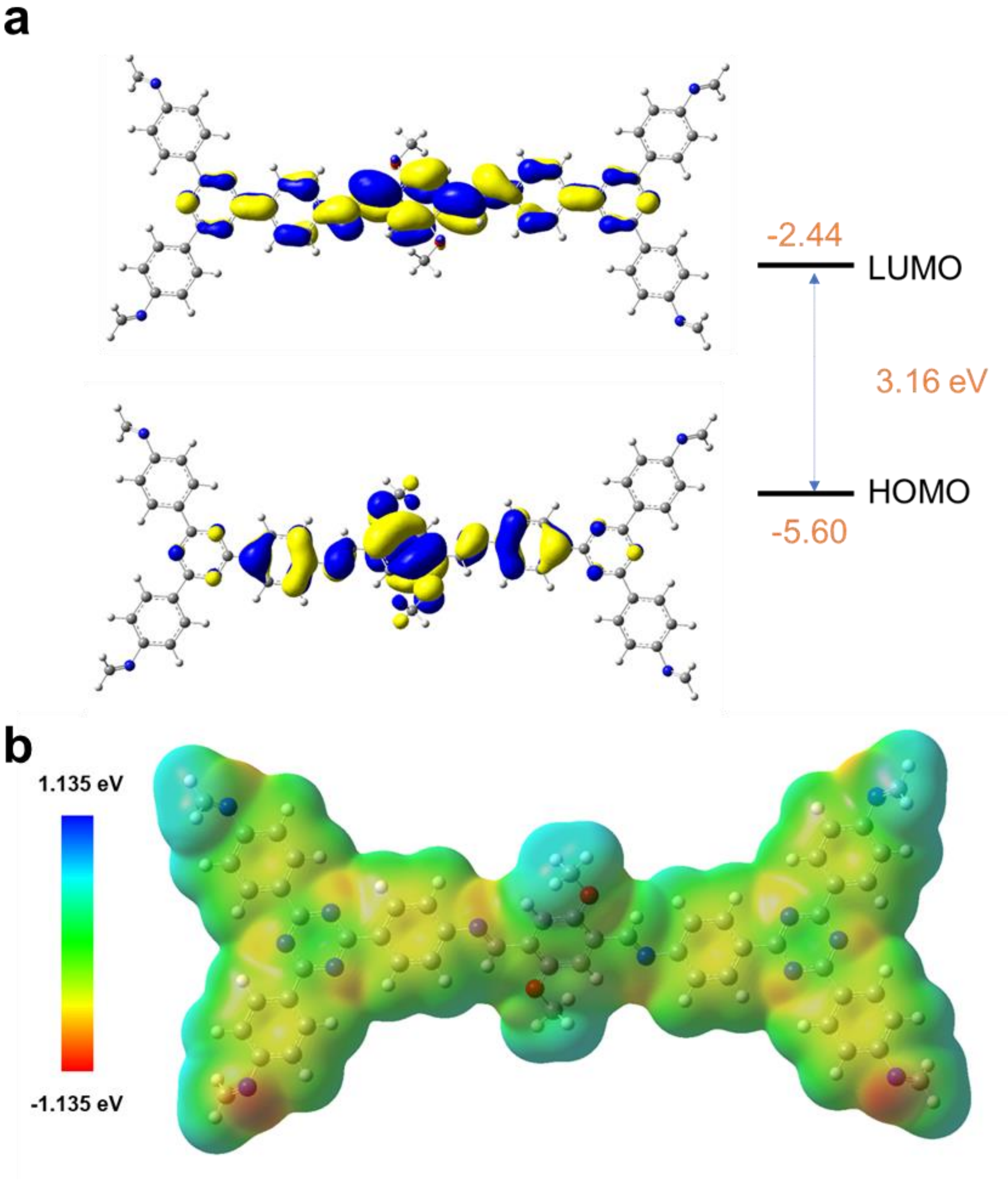

Supplementary Fig. 25. DFT calculation of the unit of TAPT-DMTA-COFs. (a) Frontier molecular orbitals and HOMO-LUMO energy level of the unit of TAPT-DMTA-COFs, (b) the molecular electrostatic potential map of the unit of TAPT-DMTA-COFs.

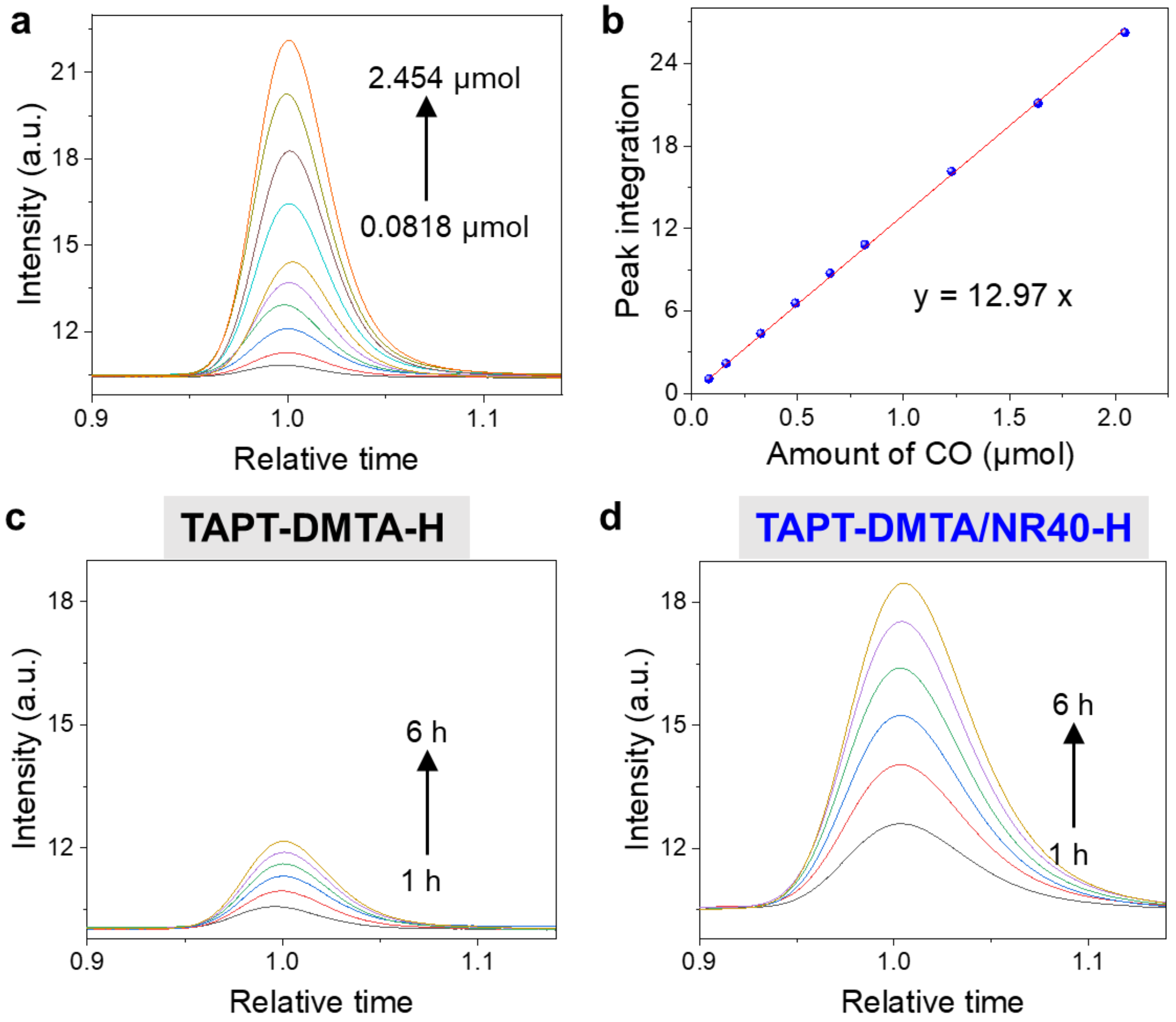

Supplementary Fig. 26. The standard curves of the CO measured based on GC. The standard curve (a,b) and catalytic data of **TAPT-DMTA-H** (c) and **TAPT-DMTA/NR40-H** (d).

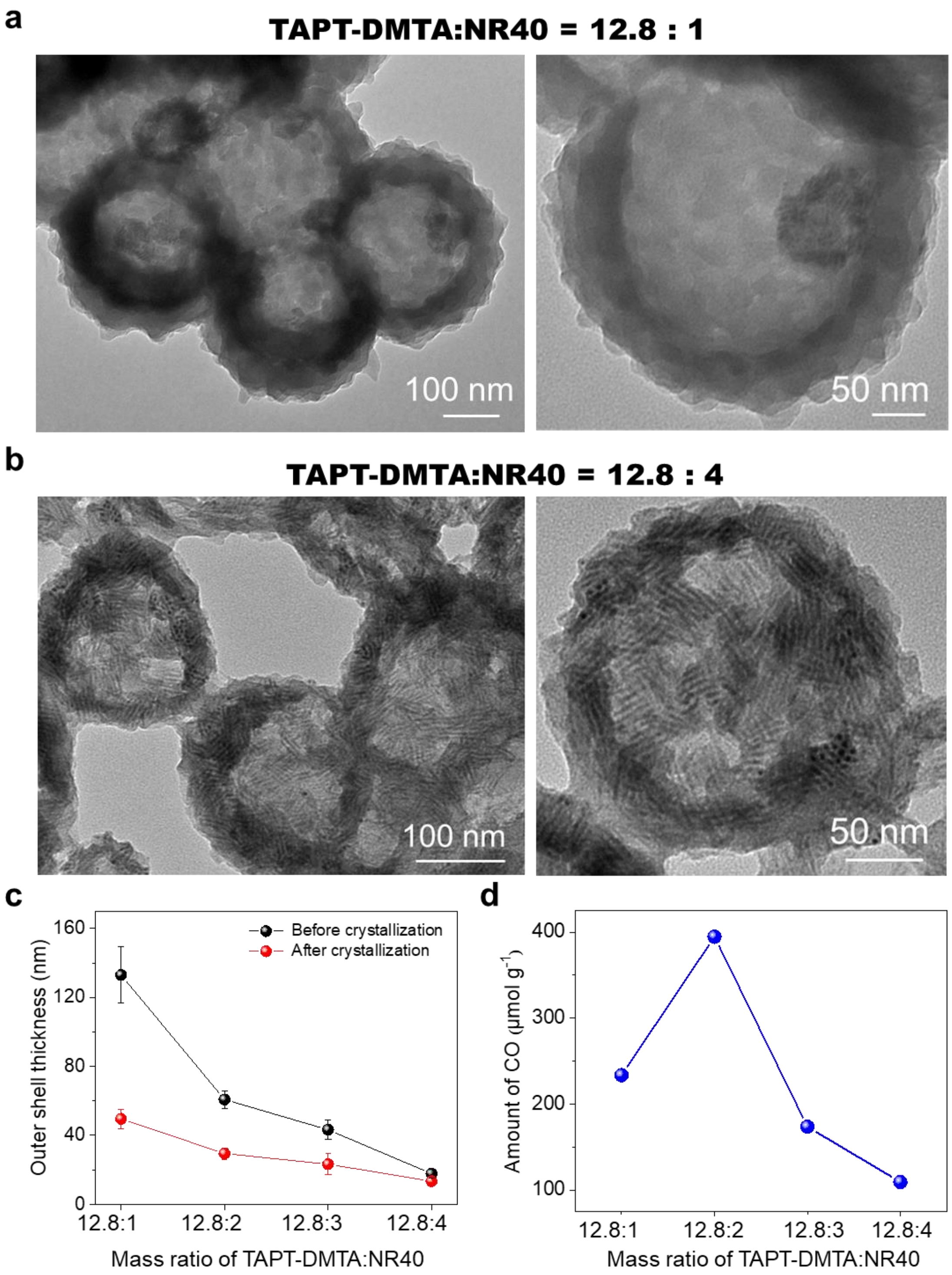

Supplementary Fig. 27. The effect of mass ratio on the performance of CO_2 photoreduction. (a) TEM images of nanocomposites prepared under TAPT-DMTA:NR40 = 12.8:1; (b) TEM images of nanocomposites prepared under TAPT-DMTA:NR40 = 12.8:4. (c) plot of the outer shell thickness over the mass ratios. (d) The CO_2-to-CO yield of different samples at different mass ratios (TAPT-DMTA:NR40).

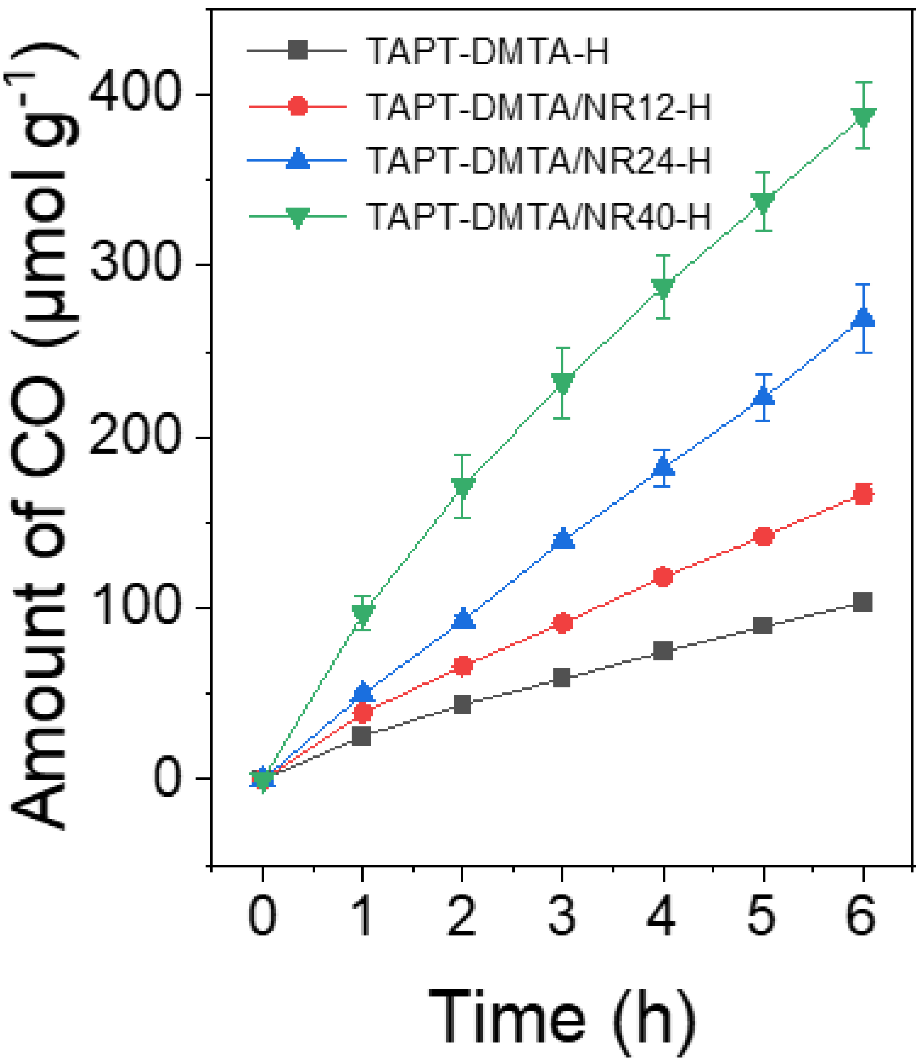

Supplementary Fig. 28. Summed CO_2 photoreduction from three independent runs. error bars were standard deviations calculated from three independent measurements.

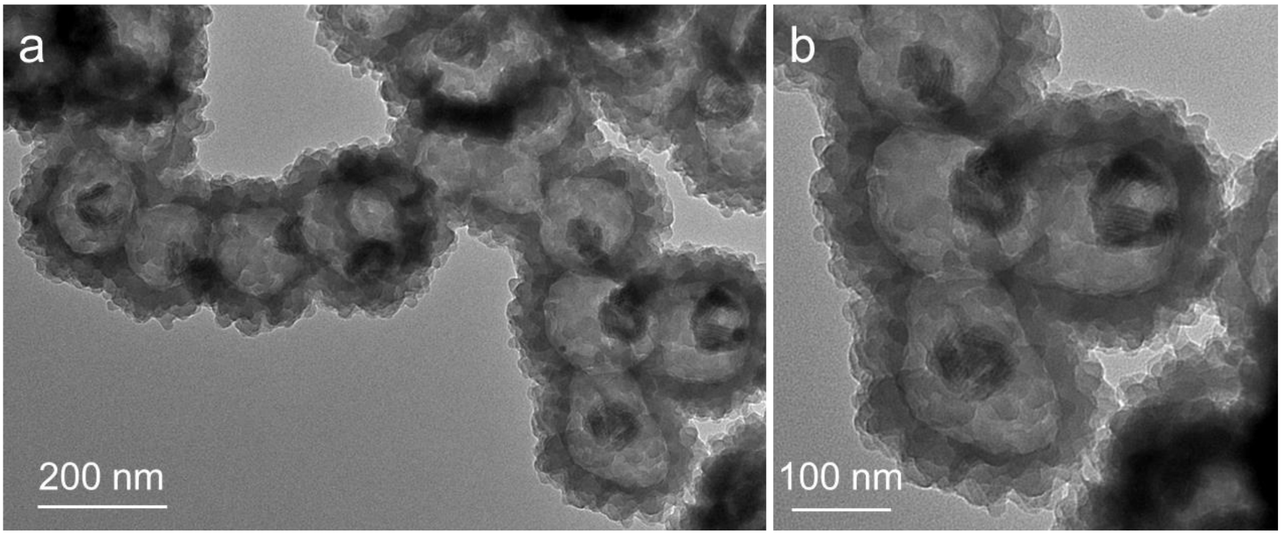

Supplementary Fig. 29. (a, b) TEM images of TAPT-DMTA/NR40-H after catalytic cycles.

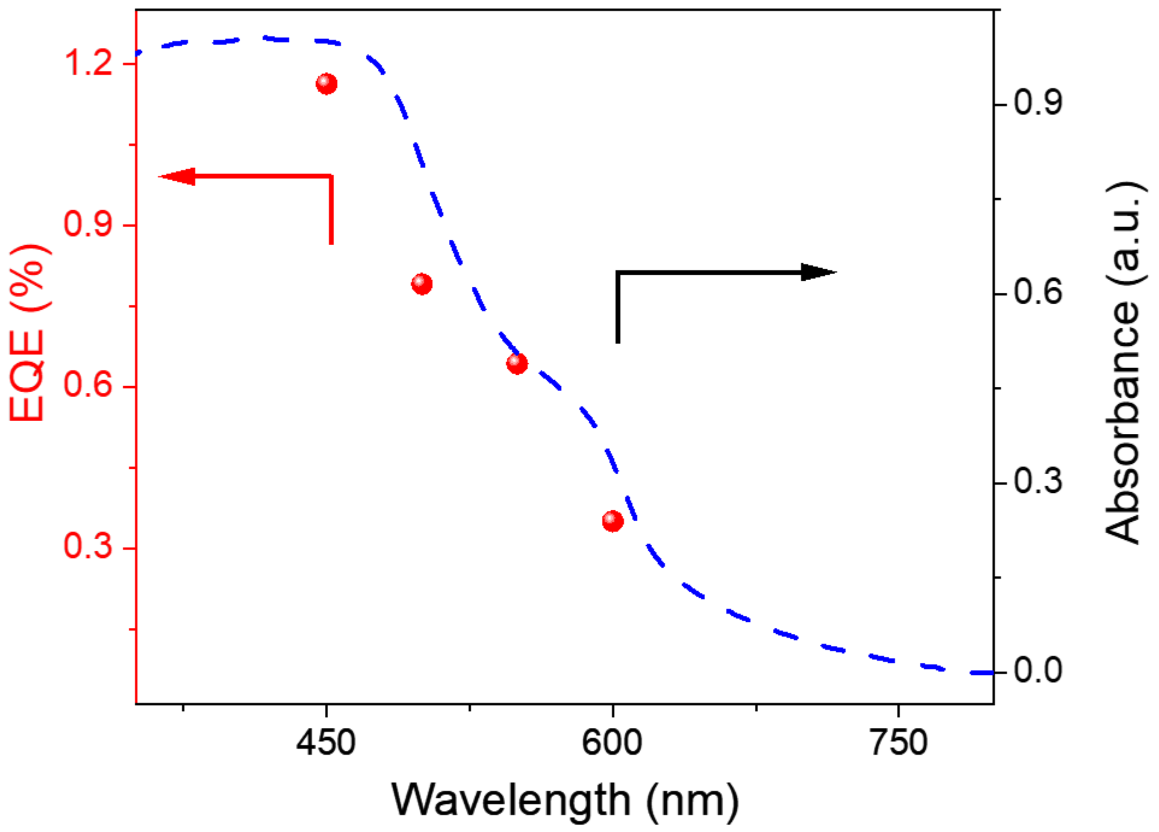

Wavelength	450 nm	500 nm	550 nm	600 nm
EQE (%)	1.16	0.79	0.64	0.35

Supplementary Fig. 30. The catalytic EQE of TAPT-DMTA/NR40-H in the presence of bandpass filter of 450, 500, 550 and 600 nm. The dashed line is the UV-vis absorbance spectrum of **TAPT-DMTA/NR40-H**. The below is the table showing the values of the EQE at various wavelengths.

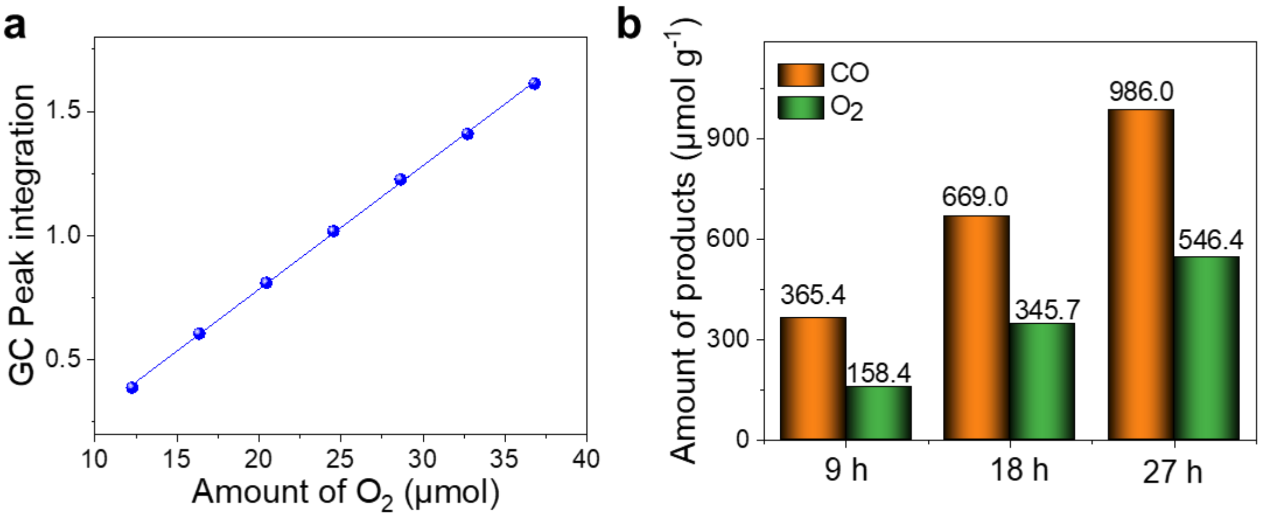

Supplementary Fig. 31. Analysis of the formed oxygen during the CO_2 photoreduction. (a) Standard curve of the oxygen gas determined by GC. (b) The quantity of CO and O_2 catalyzed by 50 mg of the **TAPT-DMTA/NR40-H** photocatalyst at different reaction times.

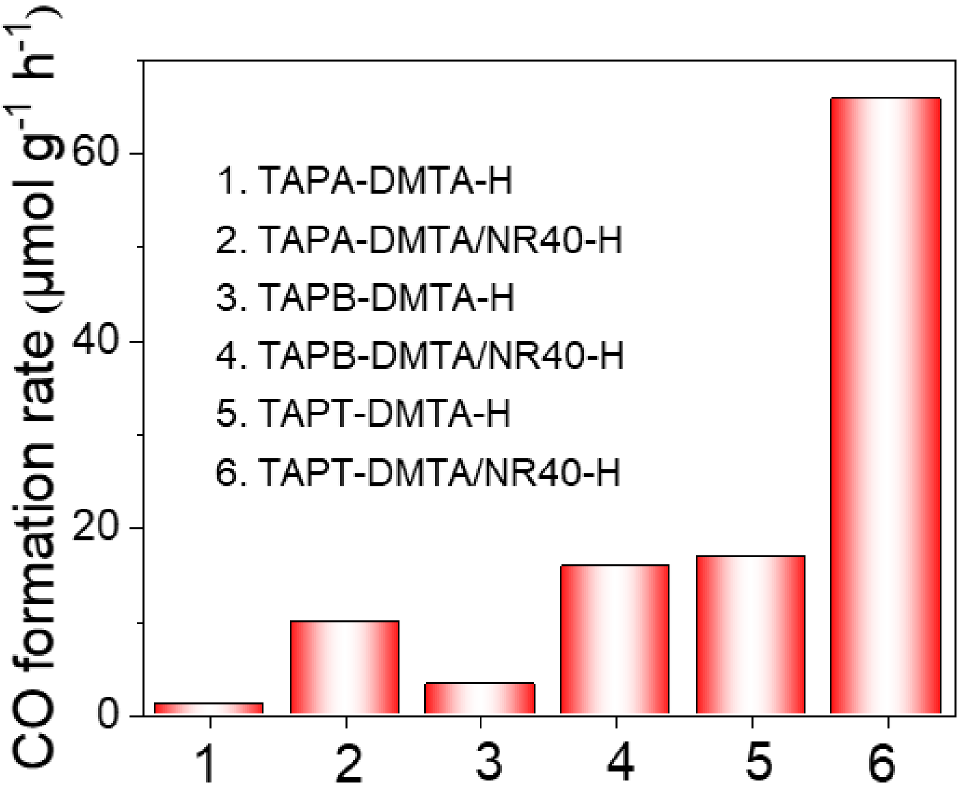

Supplementary Fig. 32. The CO_2-to-CO generation rate of different samples (replacing the TAPT monomer with TAPA or TAPB).

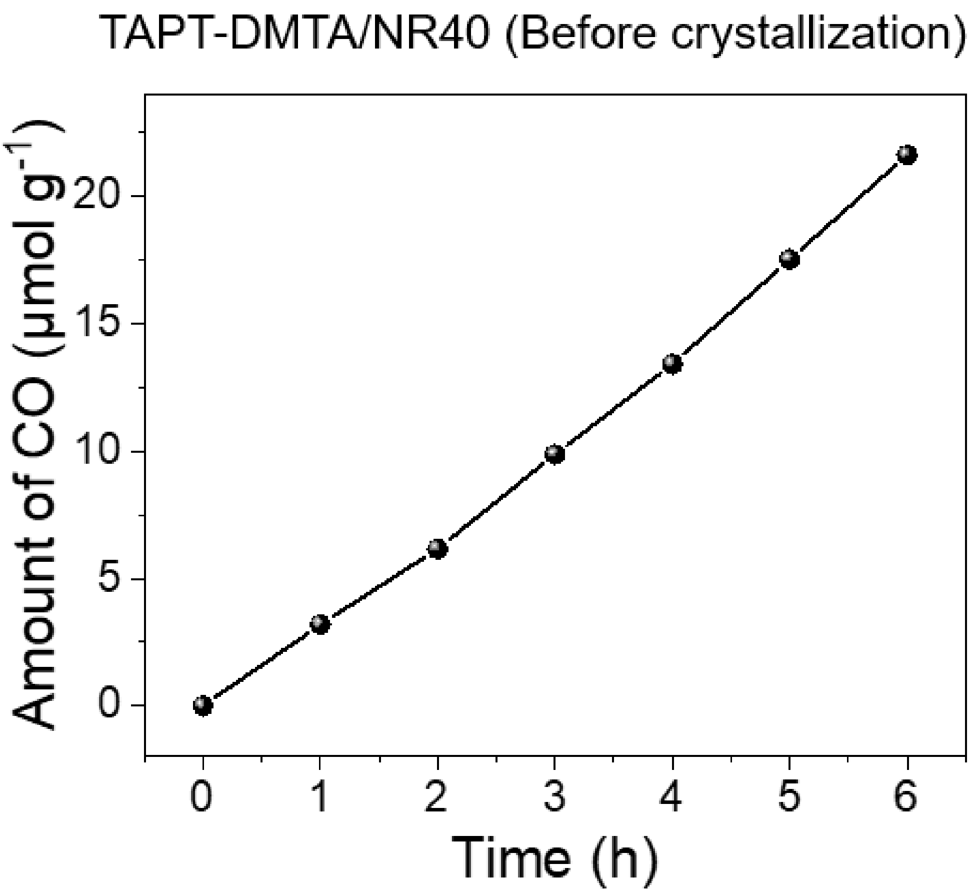

Supplementary Fig. 33. Time-dependent CO_2-to-CO performances of TAPT-DMTA/NR40 before crystallization.

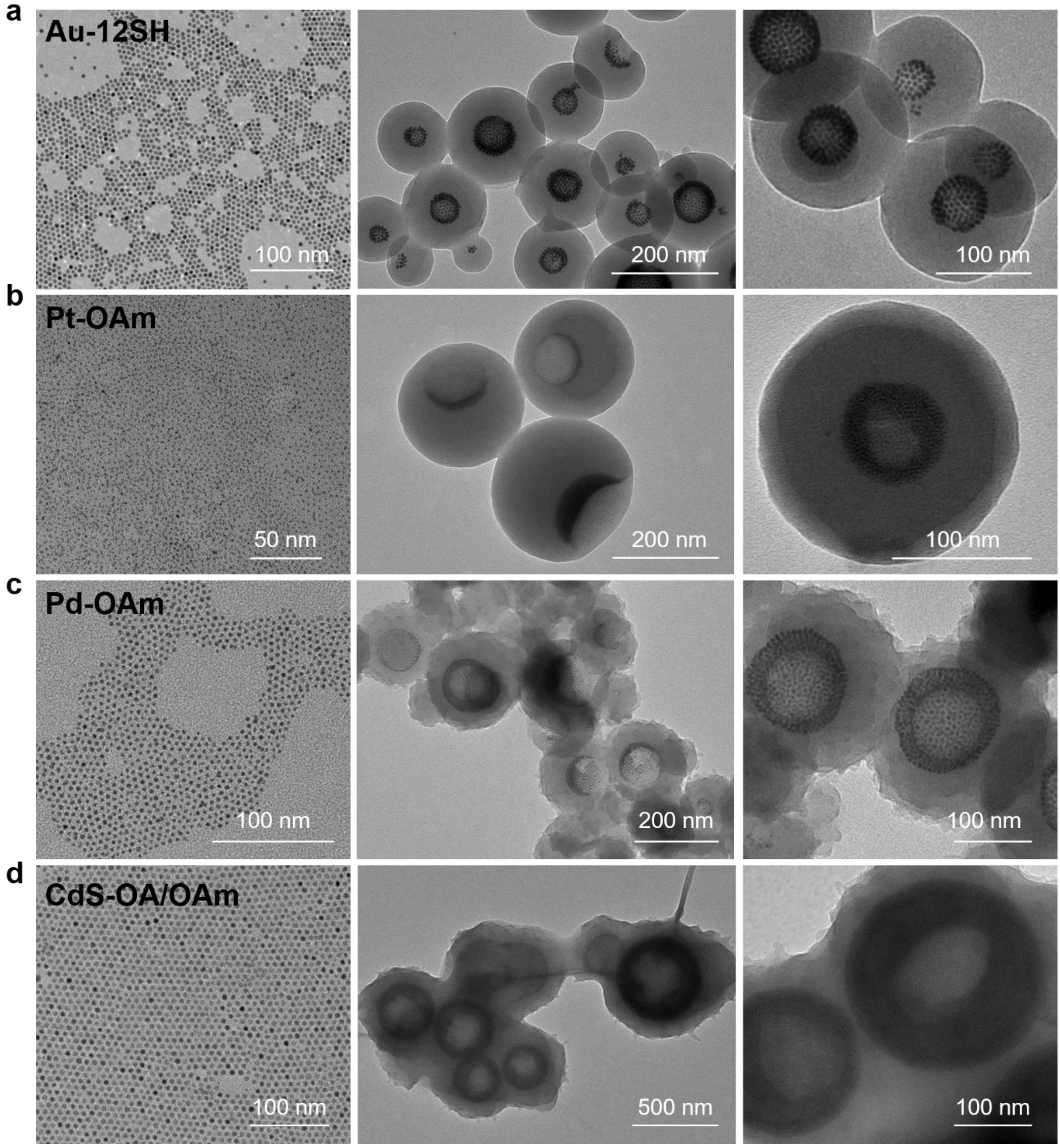

Supplementary Fig. 34. Additional morphological information of the nanocomposites. TEM images of various kinds of nanoparticles and their encapsulation into TAPT-DMTA COFs: (a) Au nanoparticles and TAPT-DMTA/Au; (b) Pt nanoparticles and TAPT-DMTA/Pt; (c) Pd nanoparticles and TAPT-DMTA/Pd; (d) CdS nanoparticles and TAPT-DMTA/CdS. The surface of Au, Pt, Pd, and CdS was coated with dodecanethiol (12SH), oleylamine (OAm), oleylamine (OAm), and oleic acid (OA) and oleylamine (OAm), respectively.

2. Supplementary Tables

Supplementary Table 1. The specific surface area, pore size and CO_2 adsorption quantity of TAPT-DMTA-S, TAPT-DMTA-H, TAPT-DMTA/NR40-S, and TAPT-DMTA/NR40-H.

Sample	Specific surface area (m^2/g)	Pore size (nm)	CO_2 adsorption quantity ($cm^3\ g^{-1}$)
TAPT-DMTA/NR40	44.53	8.8, 10.1, 13.8, 22.7	-
TAPT-DMTA-S	470.70	1.7, 2.4	4.9
TAPT-DMTA-H	622.30	1.7, 2.4	9.2
TAPT-DMTA/NR40-S	240.47	1.7, 2.4, 3.9	5.3
TAPT-DMTA/NR40-H	559.62	1.7, 2.4, 7.8, 10.6, 13.9	13.8

Supplementary Table 2 The exciton decay time of the different samples.

Sample	A_1 (%)	τ_1 (ns)	A_2 (%)	τ2 (ns)	A_3 (%)	τ_3 (ns)	$\tau_{Ave.}$ (ns)
NR40 assemblies	49.22	9.52	37.03	29.86	13.75	176.14	39.96
TAPT-DMTA-S	48.54	0.07	40.77	0.5	10.69	2.72	0.53
TAPT-DMTA-H	74.06	0.28	17.46	1.61	8.47	6.46	1.03
TAPT-DMTA/NR40-S	46.80	0.08	39.54	0.47	13.66	2.42	0.55
TAPT-DMTA/NR40-H	57.84	0.03	29.40	0.28	12.77	0.96	0.22
TAPT-DMTA/NR12-H	48.98	0.1	36.94	0.54	14.08	2.35	0.58
TAPT-DMTA/NR24-H	49.95	0.02	34.31	0.26	15.74	0.99	0.25

Supplementary Table 3. Comparison of photocatalytic activity with reported CO_2 to CO conversion based on the gas-solid systems.

No.	Ligut Source	Photocatalyst	CO (μmol g^{-1} h^{-1})	Reference
[1]	300W Xe lamp, λ>420 nm	$BiVO_4/C/Cu_2O$	3.0	*ACS Catal.* **2018** [1]
[2]	300W Xe lamp	Cu/CeO_{2-x}	1.7	*ACS Catal.* **2019** [2]
[3]	300W Xe lamp	$BiOIO_3$	17.3	*Adv. Mater.* **2020** [3]
[4]	Xe lamp, λ>800 nm	CuS	14.5	*J. Am. Chem. Soc.* **2019** [4]
[5]	300W Xe lamp	$BiOIO_3$	5.4	*Adv. Funct. Mater.* **2018** [5]
[6]	300W Xe lamp	$Bi_2O_2(OH)(NO_3)$	8.1	*Adv. Mater.* **2019** [6]
[7]	UV, 305 nm	$Cs_3Bi_2I_9$	7.8	*J. Am. Chem. Soc.* **2019** [7]
[8]	150 W Xe lamp	TiO_2/g-C_3N_4	2.0	*Appl. Catal. B* **2019** [8]
[9]	300W Xe lamp	Li_2TiO_3/TiO_2	1.7	*Appl. Catal. B* **2020** [9]
[10]	300W Xe lamp , λ>400 nm	SnS_2/SnO_2 HoMSs	4.0	*Angew. Chem. Int. Ed.* **2020** [10]
[11]	300W Xe lamp	Ni-SA-x/ZrO_2	11.8	*Adv. Energy Mater.* **2020** [11]
[12]	300W Xe lamp	red phosphorus	2.1	*J. Mater. Chem. A* **2021** [12]
[13]	300W Xe lamp	Ti_3C_2 MXene/ g-C_3N_4	5.2	*Appl. Catal. B* **2020** [13]
[14]	300W Xe lamp	g-C_3N_4/Bi_2WO_6	5.2	*J. Mater. Chem. A* **2015** [14]
[15]	400 nm LEDs	ZnSe/CdS DOR	11.3	*Adv. Mater.* **2021** [15]
[16]	Xenon lamp (200-1000 nm)	TAPBB-COF	24.6	*ChemSusChem* **2020** [16]
[17]	300W Xe lamp (380-800 nm)	COF-318-TiO_2	69.67	*Angew. Chem. Int. Ed.* **2020** [17]
[18]	300W Xe lamp (300-1200 nm)	MTCN-H (ys)	16.87	*Adv. Mater.* **2021** [18]
[19]	200W Xe lamp (AM 1.5)	QS-Co_3O_4 (ZIF-67)	46.3	*J. Am. Chem. Soc.* **2019** [19]
[20]	300W Xe lamp, λ>420 nm	TAPT-DMTA/NR40-H	64.6	This work

Supplementary Table 4. The related parameters of EQE of TAPT-DMTA/NR40-H.

Wavelength (nm)	Light intensity ($mW\ cm^{-2}$)	Illumination area (cm^{-2})	Illumination time (h)	CO (μmol)	EQE (%)
450	50	12.56	10	0.13735	1.16
500	60	12.56	10	0.12456	0.79
550	55	12.56	10	0.10211	0.64
600	60	12.56	10	0.06619	0.35

3. Supplementary Notes and Methods

Supplementary note 1. Additional experiments that confirm the chemical interaction between TAPT-DMTA COFs and NR40

In addition to the macroscopic phenomenon that dot-in-rod nanostructures interact strongly with COF, we have provided the additional evidence on the chemical interaction between TAPT-DMTA COF and CdSe/CdS NRs—the interaction between the peripherical amine group (-NH_2) of TAPT-DMTA COF and CdSe/CdS NRs.

It is well known that the primary amine could be capping agents that stabilize CdS or CdSe nanoparticles[20-22], where these amines are neutral donors that interact with Cd atoms in CdS or CdSe. To confirm the chemical interaction between CdSe/CdS NRs and the peripherical amine group of TAPT-DMTA COF, we first designed an experiment as follows (Route I in Supplementary Fig. S12): 7 mg of TAPT molecules was added into 2 mL of THF containing 2 mg of CdSe/CdS NRs (NR40). The optically clear NR colloidal solution became turbid after 8 hours, indicating that these NRs were aggregated in the presence of TAPT molecules that have three terminal amine groups, which would serve as “cross linker” resulting in the flocculation of NRs. To prove that the TAPT molecules are present in the NRs aggregates, infrared (IR) spectroscopy measurements were conducted for the samples, NRs aggregates without the addition of TAPT, TAPT, and NRs aggregates in the presence of TAPT, termed as NRs, TAPT, and NRs/TAPT, respectively (Supplementary Fig. S13a). We note that these NR aggregates were separated by centrifugation and washed with THF several times to remove the unbound TAPT molecules. Both the TAPT and NRs/TAPT samples showed IR peaks at ~1500 cm^{-1} from the C=N stretching of the triazine ring, which is not observed for the NRs in the absence of TAPT, indicating that TAPT molecules are present in the NRs/TAPT sample (Supplementary Fig. S13a). We note these NRs/TAPT aggregates could not be dissolved in any common organic solvents, indicating the formation of chemical bonding between TAPT and NRs. Unfortunately, the TAPT molecule within these NRs/TAPT aggregates could not be identified by the ^1H NMR spectroscopy, which is an important tool frequently used to characterize the surface capping agents for colloidal NPs[23]. To further confirm the chemical bonding between CdSe/CdS NRs and the peripherical amine group of TAPT-DMTA COF, we used aniline as the capping agent to interact with the surface of CdSe/CdS NRs (Route II in Supplementary Fig. S12). After adding 7 mg of aniline into

2 mL of THF containing 2 mg of CdSe/CdS NRs, the colloidal solution remained optically clear after 24 hours. These NRs were collected and washed with THF to remove the unbound aniline and were dissolved in $CDCl_3$ for ^1H NMR measurement. The NMR data in Supplementary Fig. S13b clearly showed that NMR signal assigned to the hydrogen from the aromatic ring could be observed in the NRs/aniline sample, indicating that these aniline molecules were chemically bound to the surface of NRs. We note the stabilization of these NRs in solution is mainly from the aliphatic chains from the ODPA/HPA ligands.

Overall, these additional experiments clearly showed that primary amine group ($-NH_2$) could bound to the surface CdSe/CdS NRs. Hence, we believed that the peripherical amine group of TAPT-DMTA COF could also interact with the surface of CdSe/CdS NRs, which leads to the contact between COF and CdSe/CdS atomically.

Supplementary note 2. The impact of shell thickness of the nanocomposites on the photocatalytic performances

Concerning on the thickness of the outer shell and inner shell of the nanocomposites, we have changed the mass ratios between TAPT-DMTA and NR40 during the synthesis. In the main text, under a given mass ratio of TAPT-DMTA:NR40 = 12.8:2, double shelled hollow nanocomposites could be observed, and the inner and outer shell thicknesses in TAPT-DMTA/NR40-H were determined to be 14.6 ± 3.4 and 29.5 ± 3.5 nm, respectively, by counting more than 200 particles in the TEM images. When the amount of NR40 was decreased (TAPT-DMTA:NR40 = 12.8:1), TEM image in Supplementary Fig. S27a showed that the outer shell thickness of the nanocomposites was ~50 nm, which is markedly larger than that of TAPT-DMTA/NR40-H (29.5 nm). On the contrary, when the amount of NR40 was increased (TAPT-DMTA:NR40 = 12.8:4), TEM image in Supplementary Fig. S27b showed that the nanocomposites were comprised of the single shelled hollow structure comprising the TAPT-DMTA and NR40. Hence, we conclude that the outer shell thickness could be increased to ~50 nm, but it could not be decreased to a thinner shell (Supplementary Fig. S27c).

Concerning on the inner shell of the nanocomposites that is comprised of NR40, we found that it is rather difficult to adjust the inner shell thickness. In fact, the inner shell of the nanocomposites is formed from the self-assembly of NRs. Based on the results from spherical nanoparticles, a nanoparticle monolayer could be observed, which is probably formed from the interface-templated self-assembly.

Lastly, we also studied the CO_2 photoreduction performance of the nanocomposites prepared under different ratios between TAPT-DMTA and NR40 (Supplementary Fig. S27d). For nanocomposites with a thicker outer shell of ~ 50 nm, the CO yield was determined to be 234 μmol g^{-1} after 6 hours, which is markedly lower than that of the nanocomposites with a thinner outer shell (395 μmol g^{-1}). Furthermore, for nanocomposites prepared under TAPT-DMTA:NR40 = 12.8:4, the CO yield was determined to be 109 μmol g^{-1} after 6 hours, which is also much lower than that of the double shelled nanocomposites.

Supplementary Methods

Electrochemical measurement

The electrochemical measurements were performed in 0.5 M Na_2SO_4 aqueous solution using a typical three electrode cell (equipped with a plane quartz window in front), with a Pt wire counter electrode, and an Ag/AgCl reference electrode. The curve was recorded via an electrochemical workstation (CHI 760E, Shanghai, China).

A homogeneous suspension of respective COFs was prepared by dispersing 5 mg sample in 600 μL ethanol and 5 μL Nafion (5 wt% Nafion in ethanol) were added as an additive and the mixture was mechanically sonicated for 10 minutes. The working electrodes were prepared by spreading 50 μL homogeneous suspension over the surface of Indium-Tin Oxide (ITO) glass substrate (3 cm × 1 cm) and then filmed at 60 ºC in vacuum oven. For Mott-Schottky analysis and EIS measurement, a conventional three electrodes cell was used, where COFs coated ITO served as the working electrode, Pt wire as the counter electrode and Ag/AgCl was the reference electrode. A 0.5 M Na_2SO_4 aqueous solution was used as the electrolyte and purged with argon for 30 min prior to the measurement. For Mott-Schottky experiments the partition signal was 5 mV with the frequency of 1000, 2000 and 3000 Hz. The EIS measurements were performed in dark and light excitation at 0.2 V vs. Ag/AgCl, at open-circuit voltage with AC amplitude of 10 mV in frequency range of 0.01 Hz to 105 Hz. A 300 W Xe lamp fitted with the cut-off filters (≥ 420 nm) was used as the light source for the EIS measurement. The applied potentials vs. Ag/AgCl is converted to NHE potentials using the following equations:

$$E_{NHE} = E_{\mathrm{Ag/AgCl}} + E^0_{\mathrm{Ag/AgCl}}(E^0_{\mathrm{Ag/AgCl}} = 0.199\mathrm{V}) \quad \text{(S1)}$$

The calculation of the external quantum efficiency (EQE)

The external quantum efficiency (EQE) was performed under a 300 W Xe lamp with certain monochromatic light filter. In the paper, the EQE is defined as the ratio of the photocatalytic electron consumption ($N_{electron}$) to the induced photons flux per hour (N_{photon}) within a specialized wavelength range, which can be illustrate as the followed formula:

$$\mathrm{EQE}(\%) = N_{\mathrm{electron}}/N_{\mathrm{photon}} = 2N_{(\mathrm{CO})}/N_{\mathrm{photon}} \quad \text{(S2)}$$

The calculation of $N_{electron}$ is in close association with the fact that two electrons are consumed to yield one molecule of CO.

The calculation of N_{photon} can be done as follows:

$N_{\text{photon}} = (\text{I}\times\text{A}\times\text{t})/E_{\text{photon}}$ (S3)

Where I the light intensity, A is the irradiation area which is fixed to 12.56 cm^2 in the experiments, and t is the irradiation time that is 10 hours in the experiments. The photon energy $E_{\text{photon}} = hc/\lambda$ (S4), where h is the Planck's constant, c is the speed of light and λ is the specific wavelength of the light using the desired bandpass filter. To calculate the EQE of the sample, we have equipped the light source with a bandpass optical filter (450, 500, 550 or 600 nm) during the photoreduction experiments

Hence, it can be expressed as follows:

$N_{\text{photon}} = (\text{I}\times\text{A}\times\text{t})\times\lambda/hc$ (S5)

The detailed EQE at each wavelength was calculated and summarized in Table S4 and Figure S28.

Structure simulations and X-Ray Diffraction (XRD) analysis

Structural model of **TAPT-DMTA-H** was generated using the Materials Studio 2019 software package. Firstly, the experimental XRD pattern of **TAPT-DMTA-H** was subjected to powder indexing, yielding unit cell parameters with hexagonal symmetry. Then empty unit cells were built with the above unit cell parameters. Single layer models were generated by connecting monomer and filled into the unit cells. Eclipsed (AA) stacking arrangements were constructed with the above unit cell parameters and the lattice models were geometry optimized using the Forcite module (Universal force fields, Ewald summations). The corresponding diffraction patterns were simulated in the Reflex module in Debye-Scherrer geometry for comparison with experimental data. The experimental XRD patterns agreed well with the simulations from the eclipsed (AA) layer stacking models.

Density functional theory calculations Methods

Geometry optimizations and the HOMO and LUMO energy level distribution calculation of the **TAPT-DMTA-H** material single molecule at the B3LYP/6-31G (d) level of theory were performed with Gaussian 09. The calculation of the electrostatic potential (ESP) on the molecular van der Waals surfaces of the **TAPT-DMTA-H** material single molecule at the B3LYP/6-31G (d, p) level of theory were performed with Gaussian 09.

In situ DRIFTS studies of CO_2 photoreduction

In situ DRIFTS was applied to monitor the intermediates during photocatalytic CO_2 reduction on the

surface of TAPT-DMTA/NR40-H. Bruker INVENIO R FT-IR spectrophotometer equipped with a custom built in situ diffuse reflectance cell was used for the DRIFTS study. Typically, photocatalyst (10 mg) was loaded into the reaction cell, and a background spectrum was collected under vacuum. Subsequently, the humid CO_2 which firstly passed through distilled water was introduced. The reaction cell was maintained in this state for 30 min before illumination to allow adsorption-desorption equilibrium. In situ DRIFTS spectrum was collected every 5 min during the one-hour photocatalytic experiment process.

Supplementary References

(1) Kim, C., Cho, K. M., Al-Saggaf, A., Gereige, I., Jung, H. T. Z-scheme photocatalytic CO_2 conversion on three-dimensional $BiVO_4$/carbon-coated Cu_2O nanowire arrays under visible light. *ACS Catal.* **8**, 4170-4177 (2018).

(2) Wang, M., Shen, M., Jin, X., Tian, J., Li, M., Zhou, Y., Zhang, L., Li, Y., Shi, J. Oxygen vacancy generation and stabilization in CeO_{2-x} by cu introduction with improved CO_2 photocatalytic reduction activity. *ACS Catal.* **9**, 4573-4581 (2019).

(3) Chen, F., Ma, Z., Ye, L., Ma, T., Zhang, T., Zhang, Y., Huang, H. Macroscopic spontaneous polarization and surface oxygen vacancies collaboratively boosting CO_2 photoreduction on $BiOIO_3$ single crystals. *Adv. Mater.* **32**, 1908350 (2020).

(4) Li, X., Liang, L., Sun, Y., Xu, J., Jiao, X., Xu, X., Ju, H., Pan, Y., Zhu, J., Xie, Y. Ultrathin conductor enabling efficient IR light CO_2 reduction. *J. Am. Chem. Soc.* **141**, 423-430 (2019).

(5) Chen, F., Huang, H., Ye, L., Zhang, T., Zhang, Y., Han, X., Ma, T. Thickness-dependent facet junction control of layered $BiOIO_3$ single crystals for highly efficient CO_2 photoreduction. *Adv. Funct. Mater.* **28**, 1804284 (2018).

(6) Hao, L., Kang, L., Huang, H., Ye, L., Han, K., Yang, S., Yu, H., Batmunkh, M., Zhang, Y., Ma, T. Surface-halogenation-induced atomic-site activation and local charge separation for superb CO_2 photoreduction. *Adv. Mater.* **31**, 1900546 (2019).

(7) Bhosale, S. S., Kharade, A. K., Jokar, E., Fathi, A., Chang, S. M., Diau, E. W. Mechanism of photocatalytic CO_2 reduction by bismuth-based perovskite nanocrystals at the gas-solid interface. *J. Am. Chem. Soc.* **141**, 20434-20442 (2019).

(8) Crake, A., Christoforidis, K. C., Godin, R., Moss, B., Kafizas, A., Zafeiratos, S., Durrant, J. R., Petit, C. Titanium dioxide/carbon nitride nanosheet nanocomposites for gas phase CO_2 photoreduction under UV-visible irradiation. *Appl. Catal. B* **242**, 369-378 (2019).

(9) Bi, W., Hu, Y., Jiang, N., Zhang, L., Jiang, H., Zhao, X., Wang, C., Li, C. Ultra-fast construction of plaque-like Li_2TiO_3/TiO_2 heterostructure for efficient gas-solid phase CO_2 photoreduction. *Appl. Catal. B* **269**, 118810-118819 (2020).

(10) You, F., Wan, J., Qi, J., Mao, D., Yang, N., Zhang, Q., Gu, L., Wang, D. Lattice distortion in hollow multi-shelled structures for efficient visible-light CO_2 reduction with a SnS_2/SnO_2 junction.

Angew. Chem. Int. Ed. **59**, 721-724 (2020).

(11) Xiong, X., Mao, C., Yang, Z., Zhang, Q., Waterhouse, G. I. N., Gu, L., Zhang, T. Photocatalytic CO_2 reduction to CO over Ni single atoms supported on defect-rich zirconia. *Adv. Energy Mater.* **10**, 2002928 (2020).

(12) Hu, Z., Lu, Y., Liu, M., Zhang, X., Cai, J. Crystalline red phosphorus for selective photocatalytic reduction of CO_2 into CO. *J. Mater. Chem. A* **9**, 338-348 (2021).

(13) Yang, C., Tan, Q., Li, Q., Zhou, J., Fan, J., Li, B., Sun, J., Lv, K. 2D/2D Ti_3C_2 MXene/g-C_3N_4 nanosheets heterojunction for high efficient CO_2 reduction photocatalyst: dual effects of urea. *Appl. Catal. B* **268**, 118738-118748 (2020).

(14) Li, M., Zhang, L., Fan, X., Zhou, Y., Wu, M., Shi, J. Highly selective CO_2 photoreduction to CO over g-C_3N_4/Bi_2WO_6 composites under visible light. *J. Mater. Chem. A* **3**, 5189-5196 (2015).

(15) Xin, Z. K., Gao, Y. J., Gao, Y., Song, H. W., Zhao, J., Fan, F., Xia, A. D., Li, X. B., Tung, C. H., Wu, L. Z. Rational design of dot-on-rod nano-heterostructure for photocatalytic CO_2 reduction: pivotal role of hole transfer and utilization. *Adv. Mater.* 2106662 (2021).

(16) Wang, L. J., Wang, R. L., Zhang, X., Mu, J. L., Zhou, Z. Y., Su, Z. M. Improved photoreduction of CO_2 with water by tuning the valence band of covalent organic frameworks. *ChemSusChem* **13**, 2973-2980 (2020).

(17) Zhang, M., Lu, M., Lang, Z. L., Liu, J., Liu, M., Chang, J. N., Li, L. Y., Shang, L. J., Wang, M., Li, S. L., Lan, Y. Q. Semiconductor/covalent-organic-framework Z-scheme heterojunctions for artificial photosynthesis. *Angew. Chem. Int. Ed.* **59**, 6500-6506 (2020).

(18) Zhang, M., Chang, J. N., Chen, Y., Lu, M., Yu, T. Y., Jiang, C., Li, S. L., Cai, Y. P., Lan, Y. Q. Controllable synthesis of cofs-based multicomponent nanocomposites from core-shell to yolk-shell and hollow-sphere structure for artificial photosynthesis. *Adv. Mater.* **33**, 2105002-2105011 (2021).

(19) Wang, L., Wan, J., Zhao, Y., Yang, N., Wang, D. Hollow multi-shelled structures of Co_3O_4 dodecahedron with unique crystal orientation for enhanced photocatalytic CO_2 reduction. *J. Am. Chem. Soc.* **141**, 2238-2241 (2019).

(20) Joo, J. *et al.* Generalized and facile synthesis of semiconducting metal sulfide nanocrystals. *J. Am. Chem. Soc.* **125**, 11100-11105 (2003).

(21) Boles, M. A., Ling, D., Hyeon, T., Talapin, D. V. The surface science of nanocrystals. *Nat. Mater.*

15, 141-153 (2016).
(22) Huang, X., Parashar, V. K., Gijs, M. A. M. Nucleation and growth behavior of CdSe nanocrystals synthesized in the presence of oleylamine coordinating ligand. *Langmuir* **34**, 6070-6076 (2018).
(23) Hens, Z., Martins, J. C. A solution NMR toolbox for characterizing the surface chemistry of colloidal nanocrystals. *Chem. Mater.* **25**, 1211-1221 (2013).
